# Effects of an e-health enhanced multicomponent outpatient intervention for children and adolescents with overweight or obesity in routine primary care (STARKIDS): a cluster-randomized controlled trial

**DOI:** 10.1016/j.lanepe.2026.101897

**Published:** 2026-10-14

**Authors:** Katrin Ziser, Rebecca Erschens, Stephan Zipfel, Peter Martus, Johann Jacoby, Anne Herschbach, Annalena Wels, Isabelle Mack, Alisa Weiland, Inga Krauß, Constanze Greule, Gorden Sudeck, Ansgar Thiel, Guido Zurstiege, Andreas Hoell, Hans Joachim Salize, Wolfgang Bethge, Monika Ums, Torben Sammet, Maren Reyer, Olaf Schliesing, Katrin E. Giel, Stefan Ehehalt, Florian Junne

**Affiliations:** a Department of Psychosomatic Medicine and Psychotherapy, University Hospital Tuebingen, Osianderstraße 5, 72076, Tuebingen, Germany; b German Centre for Mental Health (DZPG), Partner Site Tuebingen, 72076, Tuebingen, Germany; c Institute of Clinical Epidemiology and Applied Biometry, Medical Faculty, University of Tuebingen, Silcherstraße 5, 72076, Tuebingen, Germany; d Department of Sports Medicine, Medical University Hospital Tuebingen, Hoppe-Seyler-Straße 6, 72076, Tuebingen, Germany; e Interfaculty Research Institute for Sport and Physical Activity, Eberhard Karls University of Tuebingen, Tuebingen, Germany; f Institute of Sports Science, University of Tuebingen, Wilhelmstraße 50, 72074, Tuebingen, Germany; g German Sport University, Am Sportpark Müngersdorf 6, 50933, Cologne, Germany; h Institute of Media Studies, University of Tuebingen, Wilhelmstraße 124, 72074, Tuebingen, Germany; i Clinic for Psychiatry and Psychotherapy, Central Institute of Mental Health, Medical Faculty Mannheim, University of Heidelberg, J5, 68159, Mannheim, Germany; j German Centre for Mental Health (DZPG), Partner Site Mannheim-Heidelberg-Ulm, J5, 68159, Mannheim, Germany; k Center for Clinical Trials, Medical Faculty, University of Tuebingen, Frondsbergstraße 23, 72070, Tuebingen, Germany; l Ministry for Social Affairs, Health and Integration Baden-Württemberg, Department 7 – Health Department, Nordbahnhofstraße 135, 70191, Stuttgart, Germany; m Data Medico GmbH, Hans-Thoma-Straße 72, 69121, Heidelberg, Germany; n Public Health Department of Stuttgart, Schloßstraße 91, 70176, Stuttgart, Germany; o Department of Psychosomatic Medicine and Psychotherapy, Otto von Guericke University Magdeburg, Leipziger Straße 44, 39120, Magdeburg, Germany; p German Center for Mental Health (DZPG), Partner Site Halle-Jena-Magdeburg, Magdeburg, Germany

**Keywords:** Pediatric obesity, e-health, Routine care, Outpatient intervention, Family-focused

## Abstract

**Background:**

Pediatric obesity is a major global public health challenge, associated with increased risk for numerous sequelae, persistence into adulthood, and growing economic burden. STARKIDS is a stepwise, tailored, e-health-enhanced, family-focused intervention in primary care. It integrates nutritional counseling targeting dietary energy density reduction, physical activity promotion, behavioral strategies, and psychosocial support.

**Methods:**

We tested whether STARKIDS improves weight development and quality of life (QoL) compared with usual care. A cluster-randomized controlled trial was conducted in pediatric/family practices enrolling children/adolescents with overweight/obesity. Practices were stratified and block-randomized (no blinding) to STARKIDS or one-time counseling (optimized treatment-as-usual, TAU-O). Outcomes were assessed at baseline, 12, and 18 months. Primary outcomes were changes in BMI z-scores and QoL. Trial registration: DRKS00022813.

**Findings:**

89 practices were cluster-randomized (44 intervention, 45 TAU-O). Between April 2022 and July 2023, 379 families were assigned to STARKIDS and 232 to TAU-O. 343 and 216 families were included into the intention-to-treat analysis. After 12 months, 114 (33%) participants in the STARKIDS group and 47 (22%) in the TAU-O group achieved a BMI z-score reduction ≥0.2 (risk difference = 0.11, 95% CI 0.03, 0.20, *p* = 0.0256). After 18 months, no group differences in BMI z-score reductions were found (*p* = 0.6940). No significant differences in QoL were observed between study arms at either time point among adolescents. Among parents/guardians, a statistically significant between-group difference was observed at 18 months (−2.10, 95% CI −4.13 to −0.06; *p* = 0.044), though not considered clinically meaningful. No treatment-related serious adverse events were reported.

**Interpretation:**

STARKIDS improved BMI z-score over 12 months with effects comparable to more intensive programs. The German Federal Joint Committee recommended transferring step 1 into routine care for children/adolescents with obesity. Effects were not sustained at 18 months, indicating a need for adaptations including additional contact hours to reinforce achieved outcomes, followed by a gradual reduction.

**Funding:**

Innovation Committee of the German Joint Federal Committee (G-BA).

**Research in context**

**Evidence before this study**

We searched PubMed and PsycInfo from database inception to September 10, 2025 using the following search term: (child∗ OR adolescen∗) AND (overweight OR obesity) AND (primary care OR pediatrician OR pediatric practice OR family practice) AND (e-health OR online OR web-based OR blended) AND (intervention OR program OR therapy) with no language restrictions. There were several studies investigating multicomponent family-focused treatment options for pediatric obesity although none combined the implementation of these treatments into the pediatric primary care setting with e-health support. A Cochrane review from 2025 summarizing studies on the combination of digital interventions with conventional care found only a small sample of available studies but concluded that these interventions may have a small, short-term effect on fat mass but their effect on other outcomes and long-term clinical benefit are unclear. This is in line with existing studies on multicomponent family-focused interventions showing modest improvements in body mass index z-scores in the short term. Evidence suggests that high-intensity treatments with at least 26 contact hours are most effective. Additionally, there was a number of studies transferring existing programs in telemedicine-based formats in course of the Covid-19 pandemic that showed feasibility and acceptance as well as overall similar effects compared to in-person programs.

**Added value of this study**

Our findings support previous evidence from multicomponent intervention programs demonstrating that moderate reductions in BMI z-scores can be achieved over a one-year period and add that this can be done not only in high-intensity settings but also through a low-threshold program. Furthermore, the results show that hybrid provision and e-health augmentation of routine outpatient care in accordance with obesity prevention and treatment guidelines can successfully be implemented in pediatric and family practices into the primary care setting thus paving the way for standardized nation-wide low-threshold care in Germany. Our analyses show high satisfaction with the program among pediatric and family practices including the provision of digitally tailored educational interventions delivered by pretrained medical assistants as well as participating families.

**Implications of all the available evidence**

Digitally enhanced and tailored treatment programs to tackle childhood overweight or obesity can be implemented into pediatric primary care settings. Moderate and meaningful effects can be achieved successfully with this low-threshold intervention in outpatient routine care. Future research should address the maintenance of achieved treatment success through e.g. gradually reducing contact times instead of abruptly ending interventions. Additionally, this line of low-threshold but comprehensive multicomponent care should be continued in view of the persisting high rates of pediatric obesity and potentially extended through e.g. artificial intelligence systems allowing for further individualization.

## Introduction

Overweight and obesity in childhood and adolescence have emerged as one of the most critical public health issues globally. According to the Global Burden of Disease Study, the prevalence of overweight doubled between 1990 and 2021, while the prevalence of obesity tripled in this period.^1^ Forecasts indicate that global obesity prevalence in children and adolescents will continue to rise through 2025 for all populations in all world regions.^1^ In Germany, national surveillance data indicate that the prevalence of overweight and obesity among children and adolescents has remained relatively stable over recent decades.^2^ However, the COVID-19 pandemic was associated with a marked increase in severe obesity, particularly among older children and adolescents, which has since partially recovered in younger age groups but remains elevated in older children.^3^

This is alarming because pediatric obesity is associated with an increased risk for a multitude of sequelae, including metabolic and cardiovascular complications such as type 2 diabetes or hypertension, musculoskeletal problems such as joint pain or impaired mobility,^4^ respiratory conditions such as asthma,^5^ and psychological complications including low quality of life, low self-esteem, or depressive symptoms.^6^ It also raises the economic burden through direct healthcare costs associated with inpatient and outpatient treatment, medication and remedies and indirect costs associated with lost productivity.^7^ Considering these effects and the frequent persistence of pediatric obesity into adulthood,^8^ early preventive and therapeutic interventions are essential.

Numerous intervention programs for pediatric obesity have been developed and evaluated in recent years, ranging from individual lifestyle counseling to multicomponent behavioral family-focused interventions, school-based health promotion and, in some but in childhood rather rare cases, surgical treatments.^9^^,^^10^ Additionally, glucagon-like peptide-1 analogs such as liraglutide and semaglutide have recently shown promising effects in children aged 6 to under 12 years^11^ and adolescents aged 12 and older,^12^ respectively. However, evidence from adults suggests that stopping these medications often leads to rapid weight regain and loss of cardiometabolic benefits.^13^ This highlights the need for long-term obesity management strategies integrating pharmacotherapy, low-threshold contact points, and lifestyle-based approaches.

The best-established treatment for pediatric weight management is multicomponent interventions incorporating nutritional guidance, physical activity promotion, and behavioral strategies with a family component.^14^ Systematic reviews and meta-analyses demonstrate that these programs yield modest short-term (up to one year) improvements in body mass index (BMI) or BMI z-scores and health behaviors,^15^^,^^16^ while long-term evidence is limited.^17^ Sustainable, long-term healthy weight development and comprehensive nationwide care structures have not yet been realized,^18^ and translating interventions from specialized clinical settings into everyday life remains a major obstacle.

Two approaches currently in focus for facilitating change in everyday life are implementation in primary care and use of e-health components. Pediatric practices are the professional environment parents prefer for guidance on their child's overweight or obesity.^19^ A recent study in the US successfully implemented a family-based behavioral program in pediatric primary care with significantly better weight management than treatment-as-usual.^20^ E-health modalities such as mobile applications or web-based platforms may further facilitate transfer to everyday life through tailored information, individualized goal-setting, and feedback.^21^^,^^22^

In line with these approaches, the STARKIDS program was developed as a step-wise, tailored, e-health supported multicomponent family-focused intervention program. As a low-threshold primary care program, STARKIDS was designed to reach families at all stages of weight-related problems: children and adolescents with overweight were therefore included alongside those with obesity in line with guideline recommendations for early intervention prior to progression. It was the first of its kind to be implemented in primary care in Germany and to have its efficacy evaluated against optimized treatment-as-usual in a cluster-randomized trial. A cluster randomization was chosen to prevent contamination of the interventions in practices. We hypothesized that participants in the STARKIDS program show a greater reduction in BMI z-score and greater improvement in health-related quality of life in comparison with participants in optimized treatment-as-usual. Furthermore, the STARKIDS intervention was hypothesized to be feasible for implementation in primary pediatric care.

## Methods

### Study design

STARKIDS was a cluster-randomized controlled study with children and adolescents with overweight or obesity and one parent or legal guardian respectively. The STARKIDS study was registered with the German Clinical Trials Register on 27 November 2020, trial number DRKS00022813 (https://drks.de/search/en/trial/DRKS00022813). The trial status is closed. The study protocol has been published elsewhere.^23^ The sample size was calculated assuming a dropout rate of 25% and a cluster effect of 2, yielding a total recruitment target of 1000 children and adolescents across 100 pediatric practices. This provides ≥80% power to detect differences in the proportion of participants achieving the primary outcome (−0.2 BMI z-score reduction) of 14–17 percentage points between groups (α = 0.05, χ^2^ test adjusted for cluster effect). There was one change to the trial after it commenced concerning the planned sample: Due to challenges in recruitment during the COVID-19 pandemic and after interim analyses that revealed a significantly smaller cluster effect than anticipated, the planned sample size was reduced from 1000 to 562 families (one child/adolescent per family, i.e. individuals). A data safety and monitoring committee was implemented for the current study.

### Participants

Children and adolescents with overweight or obesity were enrolled in the study by participating pediatric practices (clusters) in the state of Baden-Wuerttemberg in Germany. Initially, eligibility criteria for participating practices consisted of enrollment in a special program of the health insurance company AOK Baden-Wuerttemberg, but were later extended to include all pediatric and family practices in the state of Baden-Wuerttemberg to facilitate recruitment. Of the 89 randomized practices, 71 enrolled families in the study. Reasons for non-enrollment of practices are detailed in Fig. 1.

**Fig. 1:**
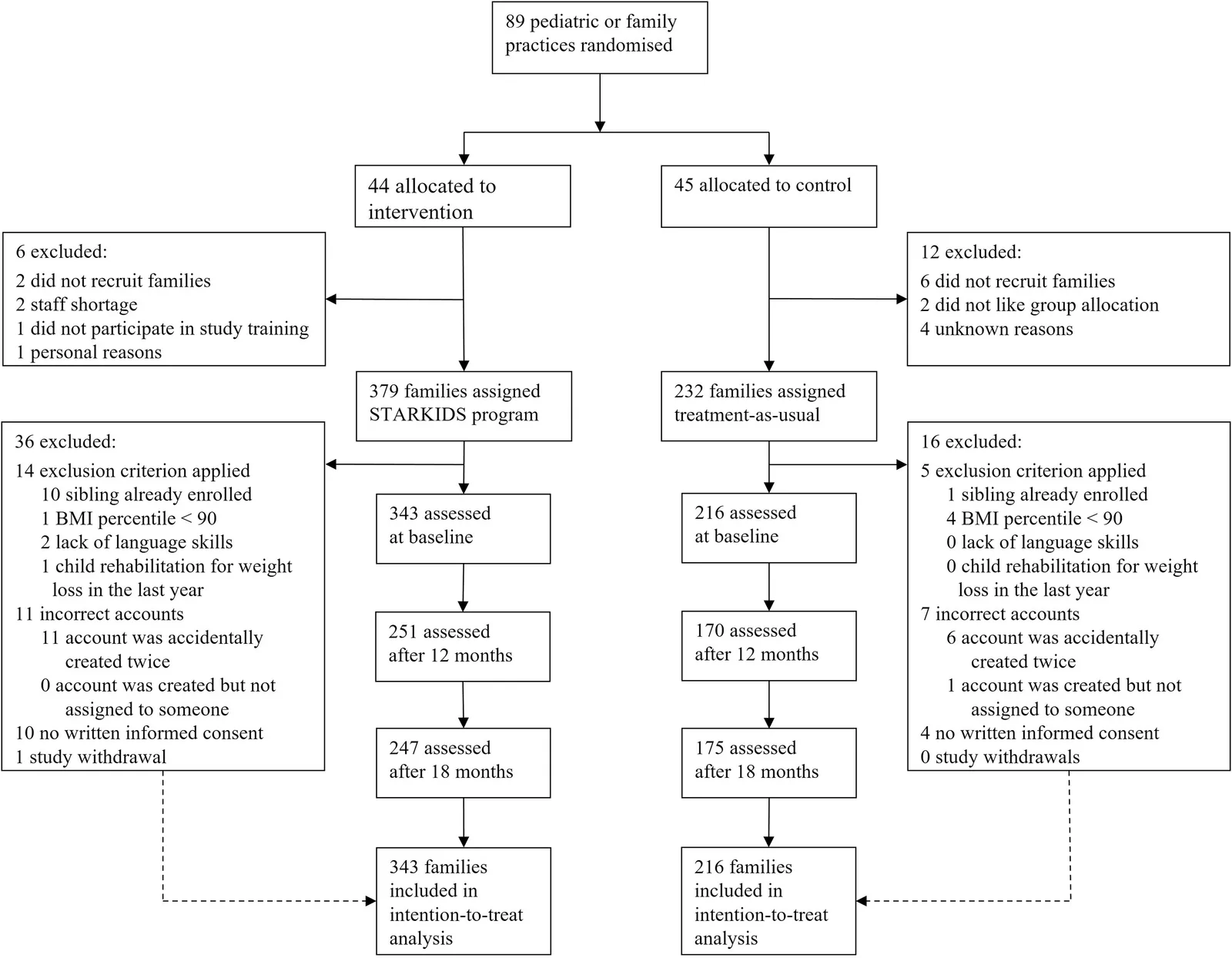
Flow of participants through the study.

Eligibility criteria for study inclusion for participating families consisted of having at least one child or adolescent between 3 and 17 years with overweight (BMI >90th percentile) or obesity (BMI >97th percentile; cutoffs according to the German guidelines^24^) and an adequate knowledge of the German language. In addition to the study protocol, the insurance requirements for participants were expanded to being insured in any statutory health insurance. Exclusion criteria included prior participation in an interdisciplinary obesity training program for one year or longer, participation in child rehabilitation or inpatient weight-loss treatment within the past year, other severe systemic diseases, and other severe psycho-social stress or disorders not associated with overweight/obesity. Written informed consent was obtained from all legal guardians and participating adolescents (12–17 years old).

Medical management of all participants, including the assessment and treatment of comorbid physical and psychosocial conditions, remained the sole responsibility of the individual primary care pediatric/family practice throughout the trial. Practices were instructed to withdraw participants from the study at any point should clinically relevant conditions such as severe eating disorders or other exclusion criteria become apparent during the intervention period, and to manage these within the framework of routine clinical care.

### Randomization and masking

The Institute for Clinical Epidemiology and Applied Biometry, Tuebingen, Germany performed the cluster-randomization of participating practices to carry out the STARKIDS program (intervention group) or one-time structured counseling (control group). The allocation sequence was randomly computer-generated. Factors for stratification of participating practices were (1) practice in an urban or rural area (according to postal code, as an indicator of socioeconomic factors) and (2) practice was a potential high recruiter or not (more than one participating pediatrician per practice or not). The program generated a series of blocks of ten for each stratum and allocated five practices to intervention group and five practices to control group randomly within each block. Due to the cluster-randomization, practices could not be blinded. Participating families were allocated through their practice after practices were randomized and trained by the study team. Allocation of families was concealed at the participant level until families consented to participate in the study.

### Procedures

Potential pediatric and family practices were recruited via flyers, network meeting presentations, the STARKIDS project website, and written invitations from the Professional Association of Pediatricians in Baden-Wuerttemberg. The study team obtained written informed consent from practices and created an online platform account for each. Practice teams of at least one pediatrician and one medical assistant were trained by the study team in the STARKIDS program or the one-time structured counseling, and subsequently recruited families through flyers and database invitations, obtaining informed consent. At baseline, pediatricians conducted physical examinations, and parents/legal guardians, and optionally adolescents aged 12 years or older, completed web-based questionnaires. Families subsequently received step 1 and, if applicable, step 2 of STARKIDS or the one-time structured counseling. Main endpoints were assessed at 12 and 18 months. A brief description of both conditions is in Panel 1 and Supplementary Figure S1 (graphical illustration), further details have been published elsewhere.^23^ Adherence was monitored via the STARKIDS online platform and individual documentation compiled locally by each practice.

Panel 1 **Treatment provided.**

**Overview**

We developed a structured framework for (secondary) preventive and interventional care for all families participating in the STARKIDS study, implemented within their regular pediatric or family practice. Trained medical assistants who were part of the families’ usual care teams conducted individually tailored, face-to-face sessions supported by digital tools. The content was based on current pediatric health guidelines and addressed key lifestyle domains known to influence healthy weight development in children and adolescents, including nutrition, physical activity, media consumption, sleep habits, family life, and stress.

**STARKIDS program**

STARKIDS is a multidisciplinary lifestyle intervention integrating nutritional counseling with a focus on reducing dietary energy density, physical activity promotion with a focus on physical activity enjoyment, behavioral strategies, and psychosocial support within routine pediatric primary care. In step 1 of the STARKIDS program, families attended face-to-face sessions administered by pre-trained medical assistants in their pediatric practice and had continuous access to an accompanying intersession e-health online platform. The medical assistants delivering the intervention were provided with a structured session manual containing suggested discussion prompts and example phrases for each interactive slide, designed to guide conversations in line with the principles of motivational interviewing.

Each face-to-face session followed a standardized sequence of components including review of weight development, motivational assessment, educational content, and collaborative goal-setting. The content within each component was individually tailored based on pre-session assessments and family-specific needs. The online platform provided access to the full content library and provided concrete implementation ideas and behavior-specific materials aligned with the goals agreed upon during the preceding face-to-face session.

The program comprised five core modules: STARKIDS Start, Eating and Drinking, Activity and Media, Family Life, and STARKIDS Keep on, as well as the optional STARKIDS Joker module with a focus on potential barriers, problems or motivational help. STARKIDS Start introduced the program, provided information about weight development in childhood and adolescence, and introduced key concepts of the following modules (e.g. meal frequency and timing, adequate amounts of physical activity, and family as a support system). It also provided an introduction to mindfulness, which was integrated into all subsequent modules. Eating and Drinking covered the dietary concept in STARKIDS and informed participants about energy density, portion sizes, tips for everyday life, and uncontrolled eating. Activity and Media covered enjoying physical activity, exploring locations for physical activity, being physically active together and in different settings, as well as media consumption. Family Life aimed to strengthen family life and inform about dealing with stress, sleeping habits, weight stigmatization, and self-worth. STARKIDS Keep on provided a reflection on the past year with the STARKIDS program as well as strategies for dealing with setbacks. Families attended individual 90-min face-to-face sessions every 3 months over the course of one year, each session covered one module in sequence. All sessions were conducted by trained medical assistants using the principles of motivational interviewing, a person-centered counseling style designed to reduce ambivalence and to strengthen intrinsic motivation and commitment to change. Each face-to-face training followed a standardized structure including a pre-assessment, a review of progress since the last session, tailored educational input on the current topic based on the results of the respective pre-assessment, and the joint individual selection of new behavioral and weight-related goals for the next three months. The e-health platform offered module-specific digital content including educative texts, short films, serious games, reflection tools, and printable tip sheets to support daily life changes. Families were expected to engage with these materials and (upon consent) received biweekly reminders via text message to monitor their individually selected goals and the child's weight.

After completing step 1 (12 months), families were evaluated based on predefined BMI z-score reduction criteria. Families whose children/adolescents had not achieved sufficient BMI z-score reduction were offered step 2, which consisted of 1–2 targeted counseling sessions delivered by public health services within a 6-months timeframe to direct the respective families in further need to local specialized services. All families continued to use the STARKIDS online platform for this timeframe.

**One-time structured counseling (optimized treatment-as-usual)**

In pediatric practices assigned to the control group, families received a single 30-min face-to-face structured counseling session delivered by a trained medical assistant. The session covered the same core topics as the STARKIDS program. However, it was limited to the provision of guideline-based recommendations and did not offer any additional behavioral tools or follow-up. At the end of the session, families received a printed flyer summarizing the recommendations. Importantly, this structured counseling was provided in addition to usual care. Families continued to have access to all regular pediatric visits, routine health checkups, and any other ongoing medical or psychosocial treatments as part of standard care. Families could also receive treatment for overweight obesity, beside inpatient treatment and treatments lasting one year or longer (exclusion criteria of the STARKIDS study).

### Outcomes

All outcomes were measured at baseline (T0), after 12 months (T1) and after 18 months (T2) on the individual participant's level. All self-reported outcomes for participating children/adolescents were gathered from parents/legal guardians and additionally, when applicable, directly from adolescents aged 12 and older. Weight development and self-reported health-related quality of life were the primary outcomes of the STARKIDS study. Weight and height were measured by practice staff at the pediatric/family practice at T0, T1 and T2, and weight development was assessed using BMI standard deviation equivalents (BMI z-scores, derived according to the LMS method^25^ and norms for a representative German reference population^26^^,^^27^). The intervention was considered successful if a reduction of at least 0.2 BMI z-score was achieved, a threshold considered to represent a clinically meaningful difference.^28^ Health-related quality of life was measured with the web-based questionnaire KINDL^R,^^29^ as a positive change of mean in childrens'/adolescents' quality of life from T0 to T1 and T2. Raw values were transformed to a scale of 100 according to established algorithms by the KINDL-group with higher mean values indicating better quality of life. KINDL scores were available from the parental perspective for all participants and from the self-report perspective for adolescents aged 12 years and older.

Secondary outcomes consisted of demographic, physiological and psychological variables such as incidences of somatic diseases related to overweight and motivation. Sonographic exams and blood levels were optional. Usage data was collected via the STARKIDS online platform (e.g. click frequencies). Outcomes related to components of the intervention, the health economic evaluation and process evaluation were gathered via web-based questionnaires. Additionally, a qualitative process evaluation was conducted via qualitative interviews. A full list of secondary outcomes can be found in the study protocol.^23^ The present reporting on the STARKIDS study focuses on the primary endpoints and feasibility data (e.g. usage, process evaluation), for an overview of feasibility-related secondary outcomes, see Supplementary Table S1. Detailed reporting of secondary outcomes across domains will be presented in dedicated separate manuscripts currently in preparation by the respective domain expert groups.

### Statistical analysis

The primary analysis was performed using a chi-square test that accounted for clustering combined with methods for the analysis of imputed data (Rao Scott method, Enders, Rubin).^30^, ^31^, ^32^ Secondary analyses of the primary endpoint were conducted using logistic regression models with generalized estimation equations (GEE) to account for cluster effects and the nesting of different measurements in participants based on difference values. Analyses were adjusted for baseline level at T0. Models with interaction terms were used to test whether subgroups with different intervention effectiveness can be identified. For health-related quality of life, further regression models were applied according to the scaling and distribution of the dependent variables, taking into account the cluster structure through random intercepts. In principle, the analyses were performed as intention-to-treat analyses.

Missing values of BMI z-scores at T0, T1, or T2 were replaced by multiple imputation (m = 500), assuming data were missing at random. Predictors for imputation included BMI z-score values of children/adolescents at T0, T1, and T2, net household income, group (intervention/control), practice ID, weight of parent/legal guardian 1, parental school leaving certificates, and migration background of parent/legal guardian 2, selected via stepwise regression from a larger candidate set (they remained in at least one of the models for predicting the measurements at T0, T1, and T2). Within each of the complete data sets created by imputation, a χ2 value was calculated in each case to determine the relationship between treatment (vs control group) and the reduction in BMI z-score by at least 0.2 (from T0 to T1 or from T0 to T2 respectively). These χ2 values were corrected for the dependence of the observation due to nesting in practices/clusters according to the method of Rao & Scott.^30^ The individual Rao-Scott-corrected χ2 values from the imputations were integrated according to Enders method.^31^ Additionally, to obtain a more comprehensive picture of BMI z-score development over time, we analyzed the development of BMI z-scores across T0, T1 and T2 using GEE. As a sensitivity analysis, we repeated the primary analysis using complete case data as missing outcome data had been imputed in the main analysis.

For the secondary outcome health-related quality of life (KINDL^R^) total scores, linear mixed models were estimated (dependent variable: difference T1-T0/T2-T0, predictors: measurement at T0, treatment group, interaction measurement at T0 × treatment group, and random intercepts for the cluster variable practice), integrated over 500 data sets with multiply imputed missing values. All analysis were performed using R (version 4.5.1).

### Ethics approval

Ethical approval of this study was obtained from the ethics committee of the Medical Faculty of the University of Tuebingen (no. 620/2020BO1) and the ethics committee of the medical association of the state of Baden-Wuerttemberg (no. B-F-2020-160). This study was conducted in accordance with the ethical principles of the Declaration of Helsinki.

### Role of the funding source

The current project was funded by the Innovation Committee of the German Joint Federal Committee (G-BA), funding number 01NVF18013 (STARKIDS). The funder of the study had no role in study design, data collection, data analysis, data interpretation, or writing of the report.

## Results

Between April 1, 2022 and July 31, 2023, 71 pediatric and family practices enrolled 611 families into the study and created accounts for them in the online platform. The number of families enrolled per practice ranged from 1 to 24. 559 underwent baseline assessments and subsequently started the STARKIDS program or optimized treatment-as-usual. Finally, 421 and 422 families were assessed for the primary endpoints after 12 and 18 months respectively. For an overview of the study flow, see Fig. 1. Overall dropout after 12 and 18 months was 25%. No serious adverse events attributable to weight loss or trial participation were reported during the study (for an overview of possible (severe) adverse events, see Supplementary File 1).

As shown in Fig. 1, participant numbers were unequally distributed between study arms, with more participants enrolled in the STARKIDS group than the TAU-O group. This imbalance may reflect two factors: more practices were excluded from the TAU-O group than from the STARKIDS group following randomization, leaving fewer active control practices, and STARKIDS practices may have shown greater recruitment engagement, possibly reflecting a perception among practice staff that they were offering an additional benefit to their patients.

Overall, mean BMI percentile of children and adolescents at baseline was 98.5 (SD 1.7). 36% of participants were in the severe obesity range, 47% were in the obesity range and only 17% were in the overweight range (see Supplementary Figure S2). Vegetative abnormalities, comorbidities and secondary diseases to obesity occurred, about one third of the sample reported either snoring (34%), concentration problems (34%), headaches (31%), Genu valgum (“knock-knees”; 28%) or foot deformities (36%). Notably, 64 participants of the 386 participants that had a sonographic exam (17%) showed indications for metabolic dysfunction-associated steatotic liver disease (MASLD). All baseline characteristics separated according to study group are in Table 1.

**Table 1:** Baseline characteristics of participating children and adolescents and their parents/legal guardians.

Individual level	STARKIDS program (n = 343)		Optimized treatment-as-usual (n = 216)	
	M (SD) or %	Median [IQR]	M (SD) or %	Median [IQR]
Age (years)	10.6 (3.1)		10.9 (3.1)	
Sex				
Female	152 (44%)		95 (44%)	
Male	191 (56%)		121 (56%)	
BMI percentile	98.5 (1.6)	99.2 [97.87–99.69]	98.3 (1.9)	99.0 [97.82–99.70]
BMI z-score	2.41 (0.49)		2.37 (0.51)	
Migration background				
Yes	178 (52%)		69 (32%)	
No	130 (38%)		125 (58%)	
Unknown	35 (10%)		22 (10%)	
Vegetative history, comorbidities and secondary diseases^a^				
Snoring	119/328 (36%)		61/209 (29%)	
Daytime sleepiness	59/328 (18%)		52/209 (25%)	
Concentration problems	108/328 (33%)		75/209 (36%)	
Hip pain	16/328 (5%)		18/209 (9%)	
Knee joint pain	59/328 (18%)		37/209 (18%)	
Headaches	105/328 (32%)		61/209 (29%)	
Abdominal pain	83/328 (25%)		58/210 (28%)	
Back pain	65/328 (20%)		36/209 (17%)	
Chronic illness with immobility	3/328 (1%)		3/209 (1%)	
Acanthosis nigricans	28/328 (9%)		11/210 (5%)	
Vision deterioration	37/328 (11%)		25/210 (12%)	
CNS deficits	1/328 (0%)		2/210 (1%)	
Vegetative disorders	8/328 (2%)		24/210 (11%)	
Hirsutism	9/328 (3%)		4/210 (2%)	
Premature adrenarche	11/328 (3%)		5/210 (2%)	
Genu valgum	86/328 (26%)		67/210 (32%)	
Foot deformity (flat feet, fallen arches, or splay feet)	110/328 (34%)		81/210 (39%)	
Spinal deformity (scoliosis)	19/328 (6%)		4/210 (2%)	
Sonographic results^a^				
Indications of metabolic dysfunction-associated steatotic liver disease (MASLD)	38/228 (17%)		26/158 (16%)	
Gallstone	1/228 (0%)		1/158 (1%)	
Hepatosplenomegaly	12/228 (5%)		15/158 (9%)	
Blood levels				
Free thyroxine (FT4); pmol/L		14.4 [13.6–15.83]		14.2 [12.58–15.58]
Total cholesterol; mmol/L		9.1^b^ [8.05–10.21]		9.0^b^ [8.10–10.27]
HDL cholesterol; mmol/L		2.6 [2.22–2.94]		2.6 [2.28–2.94]
LDL cholesterol; mmol/L		5.6 [4.88–6.6]		5.7 [4.95–6.81]
Triglycerides; mmol/L		1.0 [0.74–1.52]		1.0 [0.74–1.46]
Fasting glucose; mmol/L		4.6 [3.89–5.05]		4.6 [4.27–5.12]
Thyroid-stimulating hormone (TSH); mlU/L		2.2 [1.62–2.8]		2.3 [1.7–2.98]
Hemoglobin A1c (HbA1c); %		5.4 [5.2–5.6]		5.4 [5.2–5.5]
Alanine aminotransferase (ALT); U/L		24.0 [19.25–32]		23.5 [19–32]
Aspartate aminotransferase (AST); U/L		26.0 [22.25–31]		27.5 [22–33.25]
Study-accompanying legal guardian				
Mother	277 (81%)		175 (81%)	
Father	38 (11%)		18 (8%)	
Stepparent	1 (0%)		1 (0%)	
Grandparent	5 (1%)		1 (0%)	
Adoptive parent	2 (1%)		0 (0%)	
Other	1 (0%)		0 (0%)	
Unknown	19 (6%)		21 (10%)	
Age (years) parent/legal guardian	40.5 (6.8)		40.8 (6.7)	
BMI (kg/m^2^) parent/legal guardian	29.7 (6.6)		30.1 (6.3)	
Educational level parent/legal guardian				
No school-leaving certificate	14 (4%)		4 (2%)	
Special education leaving certificate	0 (0%)		2 (1%)	
Lower secondary school certificate	80 (23%)		49 (23%)	
Intermediate secondary school certificate	130 (38%)		79 (36%)	
Upper secondary school certificate	84 (25%)		51 (24%)	
Other school-leaving certificate	14 (4%)		10 (5%)	
Unknown	21 (6%)		21 (10%)	
Net household income				
<1000€	17 (5%)		10 (5%)	
1000–1999€	67 (20%)		23 (11%)	
2000–2999€	86 (25%)		58 (27%)	
3000–3999€	76 (22%)		46 (21%)	
4000–4999€	53 (15%)		39 (18%)	
>5000€	25 (7%)		17 (8%)	
Unknown	19 (6%)		23 (11%)	
**Cluster level**				
No. of clusters	38		33	
No. of families, mean (SD)	9.03 (4.36)		6.55 (4.10)	
Range no. of families	1–24		1–14	

BMI, body mass index; M, mean; SD, standard deviation.

a Data not available for all randomized patients.

b Out of normal range.

The sample of study-accompanying legal guardians consisted predominantly of mothers. The majority had attained an intermediate level of education, ranging from lower to upper secondary school certificate. Mean BMI among parents and legal guardians was elevated, falling in the overweight range and extending into obesity. This is consistent with the well-established role of parental overweight and obesity as a relevant risk factor for childhood obesity.

After 12 months (T1), 83/251 (33%) participants of the intervention group and 37/170 (22%) participants of the control group with measurement of BMI z-scores at T1 achieved the pre-defined criterion of BMI z-score reduction of at least 0.2. The proportion of cases across imputations was 33% in the intervention group and 22% in the control group (risk difference (RD) = 0.11, 95% CI 0.03, 0.20, *p* = 0.0256. Therefore, a significantly larger proportion of children and adolescents in the intervention group achieved a reduction of 0.2 BMI z-score or more compared to the control group. For a graphic illustration, see Supplementary Figure S3. The mean design effect (i.e. variance inflation due to clustering of participants in practices) across the multiple imputed data sets for this outcome was 1.37 (IG = 1.46, KG = 1.38).

After 18 months (T2), 71 (29%) participants of the intervention group and 48 (27%) participants of the control group achieved the pre-defined criterion of BMI z-score reduction of at least 0.2. The proportion of cases with a reduction ≥0.2 BMI z-score (from T0 to T2) via imputations was 30% in the intervention group and 28% in the control group (RD = 0.01, 95% CI −0.07, 0.10, *p* = 0.6940). The mean design effect was 1.31 (IG = 1.44, KG = 1.24). The complete case sensitivity analysis yielded results consistent with the primary analysis.

The analysis of BMI z-score development over time as a continuous outcome variable showed a greater decrease in BMI z-score between T0 and T1 in the intervention group compared to the control group (significant interaction between T1 and group, b = −0.09, 95% CI, *p* = 0.0038). There was no such effect at T2 (interaction between T2 and group, b = −0.03, *p* = 0.3873). The mean BMI z-scores in the intervention group were 2.41 (0.49) at T0, 2.24 (0.63) at T1 and 2.27 (0.62) at T2, in the control group 2.37 (0.51) at T0, 2.32 (0.59) at T1 and 2.27 (0.61) at T2. The analysis is detailed in Supplementary Table S2, a graphic illustration of the course of BMI z-scores by study group is in Fig. 2.

**Fig. 2:**
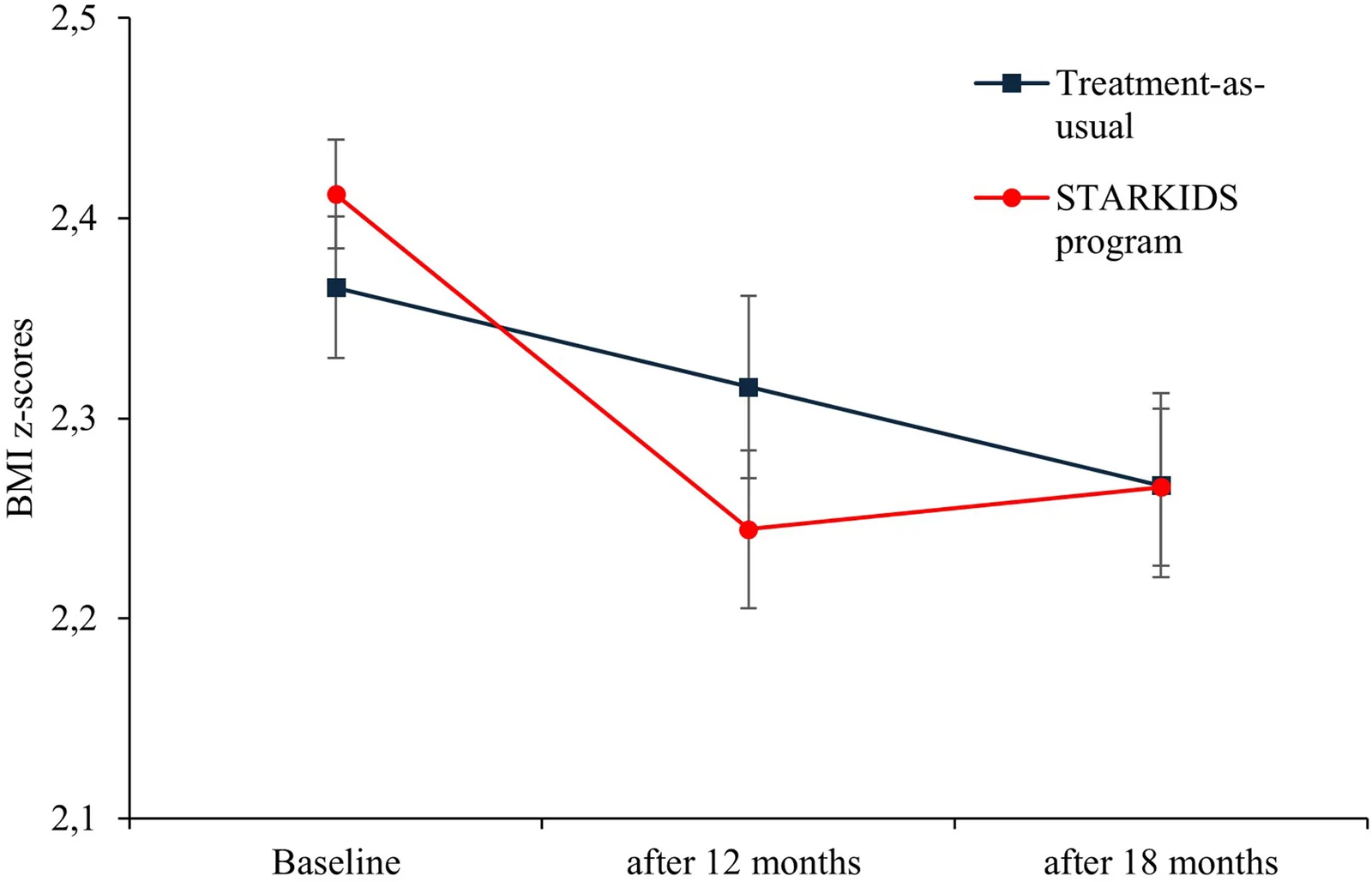
Course of BMI z-scores during the study.

The second main outcome health-related quality of life was analyzed for the assessment of the parents/legal guardians and self-assessment of adolescents respectively. Adolescents of the intervention group reported means of 64.62 (SD 14.56), 66.67 (SD 11.06) and 66.55 (SD 13.10) at T0, T1, and T2, respectively. Adolescents in the control group reported means of 65.60 (SD 13.03), 65.04 (SD 14.19) and 64.16 (SD 15.00) at T0, T1, and T2, respectively. No differences were found for the self-assessments of adolescents (*p* > 0.81 each). In case of parents and legal guardians, total scores in the intervention group decreased from a mean of 72.79 (SD 11.43) in the intervention group at baseline to 70.59 (SD 11.31) after 18 months whereas the control group remained stable from 71.71 (SD 12.12) at baseline to 71.79 (SD 12.34) after 18 months, resulting in a significant group difference between baseline and after 18 months (−2.10, 95% CI −4.13, −0.06, *p* = 0.0440).

Usage data from the online platform showed high attendance rates in both groups: 83% in the one-time structured counseling in the optimized treatment-as-usual group and 69–92% in the face-to-face sessions in the STARKIDS program. Merely the optional session STARKIDS Joker, intended for implementation difficulties, was rarely used (3%). In step 2 of the STARKIDS program, attendance was low with only 24 of 162 eligible families (15%) participating. Analysis of click frequencies showed different proportions of non-users (including dropouts) according to content type (16–53%) with mindfulness audios being the least used resource (78% non-use). There was overall good engagement with educative texts, tip sheets, and videos. Reflection tools and serious games were used less frequently. Detailed usage data are presented in Supplementary Table S3.

202/247 (82%) parents and legal guardians of the intervention group as well as 51 adolescents participated in the process evaluation at T1. Overall satisfaction with the STARKIDS program was high (parents/legal guardians: M = 4.07, SD = 0.89, adolescents: M = 3.90, SD = 0.81; scale 1–5, higher values indicate higher satisfaction). Similarly, pediatric and family practices reported high motivation (pediatricians/family doctors: M = 4.11, SD = 0.96, medical assistants M = 4.10, SD = 0.92), high enjoyment conducting the program (pediatricians/family doctors: M = 4.11, SD = 1.23, medical assistants: M = 4.07, SD = 0.94), and moderate to high feasibility (pediatricians/family doctors: M = 3.61, SD = 1.03, medical assistants: M = 3.38, SD = 1.12). 28/38 (74%) pediatricians/family doctors and 30/38 (79%) medical assistants participated in the evaluation.

## Discussion

The STARKIDS program led to significantly higher reductions in BMI z-scores in comparison to optimized treatment-as-usual after one year. However, no differences in BMI z-score development were found between the two groups after 18 months, allowing only partial confirmation of the main hypothesis. For the second main outcome health-related quality of life, no differences between groups were detected, thus not confirming our hypothesis. Usage data indicated high attendance rates of the face-to-face sessions in the STARKIDS program and optimized treatment-as-usual, while step 2 of the STARKIDS program was not accepted as indicated by very low attendance rates among those with an indication for step 2. Participating families and pediatric and family practices in the intervention group were satisfied with the STARKIDS program and its conduction.

Regarding the primary outcome of BMI z-score reduction at 12 months, the confidence interval around the observed effect is relatively wide, ranging from a small to a fairly large effect. This width, however, is in line with what would be expected given the size of our sample: even if the true population effect matched our original assumption exactly, a study of this size would still be expected to produce a confidence interval of similar width. The assumed effects were within the confidence interval of the observed one, which shows that our original sample size calculation was reasonable even so the observed effect was smaller than the assumed one.

The overall decrease of BMI z-scores is in line with other studies. Prinz and colleagues reported an average 0.23 BMI z-score reduction over 24 months in a sample of over 12.000 children and adolescents enrolled in outpatient lifestyle interventions of varying intensity across Germany, Austria and Switzerland.^28^ They also showed that a moderate loss of ≥0.2 BMI z-score -the step 1 goal in STARKIDS, achieved by almost half of their participants-has favorable cardiometabolic effects. A further German trial by Reinehr and colleagues found a BMI z-score reduction of 0.41 after one year, exceeding the STARKIDS effect, likely reflecting that program's considerably higher treatment intensity.^33^ Notably, our optimized treatment-as-usual (a one-time structured counseling session with written guideline-informed instructions) performed well enough over 18 months that no group differences remained at the final timepoint.

Against the backdrop of the COVID-19 pandemic, during which rates of pediatric overweight and obesity accelerated significantly,^34^ the observed weight improvements in both groups represent a clinically meaningful advance. During the study period (2022–2024), data from Austria and Denmark indicated that while childhood overweight prevalence returned to pre-pandemic levels, obesity prevalence remained elevated, with continued weight gain among affected children post-pandemic.^35^^,^^36^ These findings underscore the relevance of the weight trajectory improvements achieved in the current study.

Overall health-related quality of life in both groups was lower than in a German reference population, for both parent reports and adolescent self-reports.^37^ This may partly reflect lingering Covid-19 pandemic effects on adolescent quality of life, scores in STARKIDS were similar to another German study of adolescents with obesity during the pandemic.^38^ Children and adolescents with overweight and obesity have also previously shown lower quality of life across some domains than normal-weight peers.^39^ Nonetheless, our intervention did not improve health-related quality of life. The parent-reported within-group difference (T0-T2, intervention group) corresponds to only 0.14 SD of the baseline score and is well below the 0.5 SD distribution-based threshold for clinical relevance.^40^ Health-related quality of life in STARTKIDS thus seems comparable to previous pediatric obesity studies but warrants further investigation of how the intervention could better address it.

Despite promising findings for step 1 of the intervention (face-to-face sessions and online platform in pediatric/family practices), uptake of step 2 (further counseling at the local public health department) was poor. A qualitative process evaluation conducted alongside the trial (reported separately) suggested possible reasons: Families did not want to burden public health already strained by the pandemic, did not perceive them as the right setting for further counseling, received insufficient explanation of step 2 in the practice, and saw no additional value in further counseling.

Given the low participation rate in step 2, the T1-to-T2 period can be considered a follow-up period for both groups. The optimized treatment-as-usual group received no further intervention, while the STARKIDS group had access only to the online platform. Consequently, the e-health component alone may not be sufficient to maintain the achieved BMI z-scores reductions, as the STARKIDS group's advantage was not sustained between T1-T2. In line with this, a recent Cochrane review comparing digital-technology was unable to determine an effect of digital interventions alone on any reported outcome.^41^ Maintaining improvements may therefore require continued personal interactions with the pediatric/family practice.

Ways to maintain changes achieved after the first year could include adapting step 2 to provide shorter refresher sessions with the pediatric or family practice (rather than local/community health departments) to reinforce content from the face-to-face sessions, fading out over time (e.g. every 6, then 9, then 12 months). Although evidence suggests that high-intensity treatments with at least 26 contact hours are most effective,^42^ this is not always feasible in routine care. We demonstrated that our low-threshold intervention can achieve positive changes in weight development with considerably fewer contact hours. Therefore, maintaining or further improving these outcomes may be possible through a continued low-threshold approach.

Based on these findings, we propose a revised, three-step model: First, the one-time structured counseling session (originally TAU-O) becomes the new step 1, provided for all eligible patients—potentially in groups—given its reasonably favorable outcome over 18 months. Second, only families with insufficient progress after this counseling receive the STARKIDS face-to-face sessions and online platform as an intensification. Third, the original step 2, given its low acceptance, is adapted to take place in the pediatric/family practice with gradually fading contact hours and online platform access, complemented for complex cases by cross-sectoral case management from a specialized center. This approach should remain closely monitored by the practice to allow timely intervention, while remaining broadly implementable in routine care.

Strengths of this study and STARKIDS include its step-by-step approach with prolonged intervention for non-responders, suitable for routine primary care across diverse practices (urban/rural, small/large). Its content aligns with current German S3 guideline recommendations.^24^ Given the state-wide diversity of participating practices and the naturalistic sampling, the study population appears fairly representative, with a slight skew towards more participants with obesity than overweight.

We demonstrated state-wide implementation in a large sample with effectiveness on the primary endpoint shown across the overall study population after 12 months. A separately reported health economic evaluation further demonstrated cost-effectiveness for the subgroup with obesity. Based on these findings, the German Federal Joint Committee has officially recommended transfer of step 1 into routine care for this subgroup, independently supporting the program's scalability beyond the trial setting.

Due to its digital nature, the program may further be adaptable to other languages and countries. Since administration, assessments and face-to-face-session materials are entirely hosted on the online platform, quick adaptation to updated guidelines is possible. This represents a significant advantage, given shortages of specialized personnel and facilitates the integration of artificial intelligence systems that can further enhance individualization.

A further strength is the trial's relatively low attrition rate of 25% which compares favorably with rates reported elsewhere. An integrative review by Dhaliwal and colleagues of 23 family-based pediatric obesity interventions reported attrition rates of up to 83% (median 37%).^43^ This lower dropout rate may reflect the feasibility and acceptability of delivering the intervention within routine practices where families already have an established relationship with their practice team, potentially reducing barriers such as logistical obstacles and perceived relevance of treatment.

A Limitation of this study is its implementation in a routine primary care setting which was a deliberate goal but is also more susceptible to errors and bias. We mitigated this through rigorous practice training, close study-center support, and continuous monitoring, though selection bias may still be reflected in the relatively high proportion of participants with obesity vs overweight. However, no clear indications of differential effects were detected in the data. The lack of sustained effects after 18 months is unsatisfactory and indicates the program should be adapted as proposed. The 18-month observation period, while longer than the six to twelve months common in the literature, remains relatively limited and should be extended in future long-term follow-up studies. Understanding which target components (e.g. dietary energy density, physical activity, emotional well-being) drive the overall effect established here also remains an important direction for future mechanistic analyses. Finally, a comprehensive health economic analysis will be reported separately, given the complexity level of detail required for adequate reporting. In brief, it demonstrated cost-effectiveness of step 1 for the primary endpoint in the subgroup with obesity (compared with overweight and severe obesity), with a positive return on investment over a ten-year horizon.

Notably, a subset of participants was of an age at which transition from pediatric to adult healthcare becomes relevant, a critical juncture where disrupted care continuity may jeopardize treatment gains. Although not specifically designed for this purpose, STARKIDS components such as self-management skills, health literacy, and autonomous digital platform engagement align conceptually with the competencies required for a successful transition to adult healthcare. Future versions of STARKIDS might therefore consider explicitly incorporating transition-specific content for older adolescents.

In conclusion, although some aspects warrant further adaption and evaluation, our findings demonstrate the effectiveness of this low-threshold intervention over 12 months. STARKIDS achieved improvements in weight development comparable to more intensive programs. The observed effects were achieved within a multidisciplinary framework integrating energy-density–oriented nutrition, physical activity promotion with a focus on enjoyment, and behavioral and psychosocial components. STARKIDS has been successfully implemented in routine care at the state level, with effectiveness demonstrated across the overall study population. Cost-effectiveness (reported separately) was specifically shown for the subgroup with obesity for whom the German Federal Joint Committee has since recommended transfer into routine care, supporting the program's potential for nationwide scaling and accessible, evidence-based support for affected children, adolescents, and their families.

## Contributors

Funding acquisition: AHo, AT, FJ, GS, GZ, HJS, IK, IM, OS, PM, SE, SZ, TS, WB; conceptualization of the intervention: AT, CG, FJ, GS, GZ, IK, IM, KEG, KZ, SE, SZ; conceptualization & methodology: AHo, FJ, KEG, KZ, PM, SE, SZ; data curation: JJ, PM; formal analysis: JJ, PM, KZ; investigation: AHe, AWei, AWel, CG, KZ, RE, OS, TS, MR; project administration: KZ, RE; resources: FJ, KEG, PM, SZ; software: OS; supervision: FJ, KZ, RE, SE; validation: JJ, MU, PM, WB; visualization: JJ, KZ; writing—original draft: KZ; writing—review & editing: all authors. JJ and PM accessed and verified the underlying data. All authors had full access to the data in the study and approved the final version of the manuscript. KZ, SE and FJ had final responsibility for the decision to submit the manuscript for publication.

## Data sharing statement

Individual participant data that underlie the results reported in this article, after de-identification (text, tables, figures, and appendices) will be shared following publication with researchers who provide a methodologically sound proposal, e.g. for individual participant data meta-analysis. Proposals should be directed to the corresponding author. A written and signed data access agreement will be required.

## Declaration of interests

KEG reports funding from the EU grant eprObes (prevention of childhood obesity); honoraria for lectures on obesity and binge eating disorder; participation on several Data Safety Monitoring/Advisory Boards for studies on eating disorders; and unpaid leadership roles as General Secretary of the German College for Psychosomatic Medicine and Vice President of the German Society for Eating Disorders. SZ reports royalties as Editor of *Psychotherapy and Psychosomatics* (Oxford University Press) and an unpaid role as Board Member of *Lancet Psychiatry*. RE reports travel support related to the STARKIDS project, paid to the institution/project rather than to the individual. All other authors declare no competing interests.

## Appendix A Supplementary data

Supplementary Material

German Abstract

Study Protocol
