# Supplementary material for: Effects of an e-health enhanced multicomponent outpatient intervention for children and adolescents with overweight or obesity in routine primary care (STARKIDS): a cluster-randomized controlled trial
Source: Lancet Reg Health Eur. 2026 Oct 14;71:101897. doi: 10.1016/j.lanepe.2026.101897 (PMC13694850; doi:10.1016/j.lanepe.2026.101897)
Supplement: Supplementary file 1 [file mmc1.pdf]

Effects of an e-health enhanced multicomponent outpatient intervention for children and adolescents with overweight or obesity in routine primary care (STARKIDS): a cluster-randomized controlled trial

Supplementary material

Table of contents

Supplementary figure 1: Examples illustrating the graphical appearance of the STARKIDS intervention 2
Supplementary figure 2: Weight class affiliation at the three measurement timepoints by group 4
Supplementary figure 3: Proportion of participants with ≥ 0·2 BMI z-score reduction by group 5
Supplementary table 1: Feasibility-related secondary outcomes reported in the current manuscript 6
Supplementary table 2: Coefficients for GEE with BMI z-score as a continuous outcome variable 7
Supplementary table 3: Attendance rates and usage data of the STARKIDS program 8
Supplementary file 1: (Serious) Adverse Events 9

Supplementary figure 1: Examples illustrating the graphical appearance of the STARKIDS intervention

A: Overview of the step-wise program

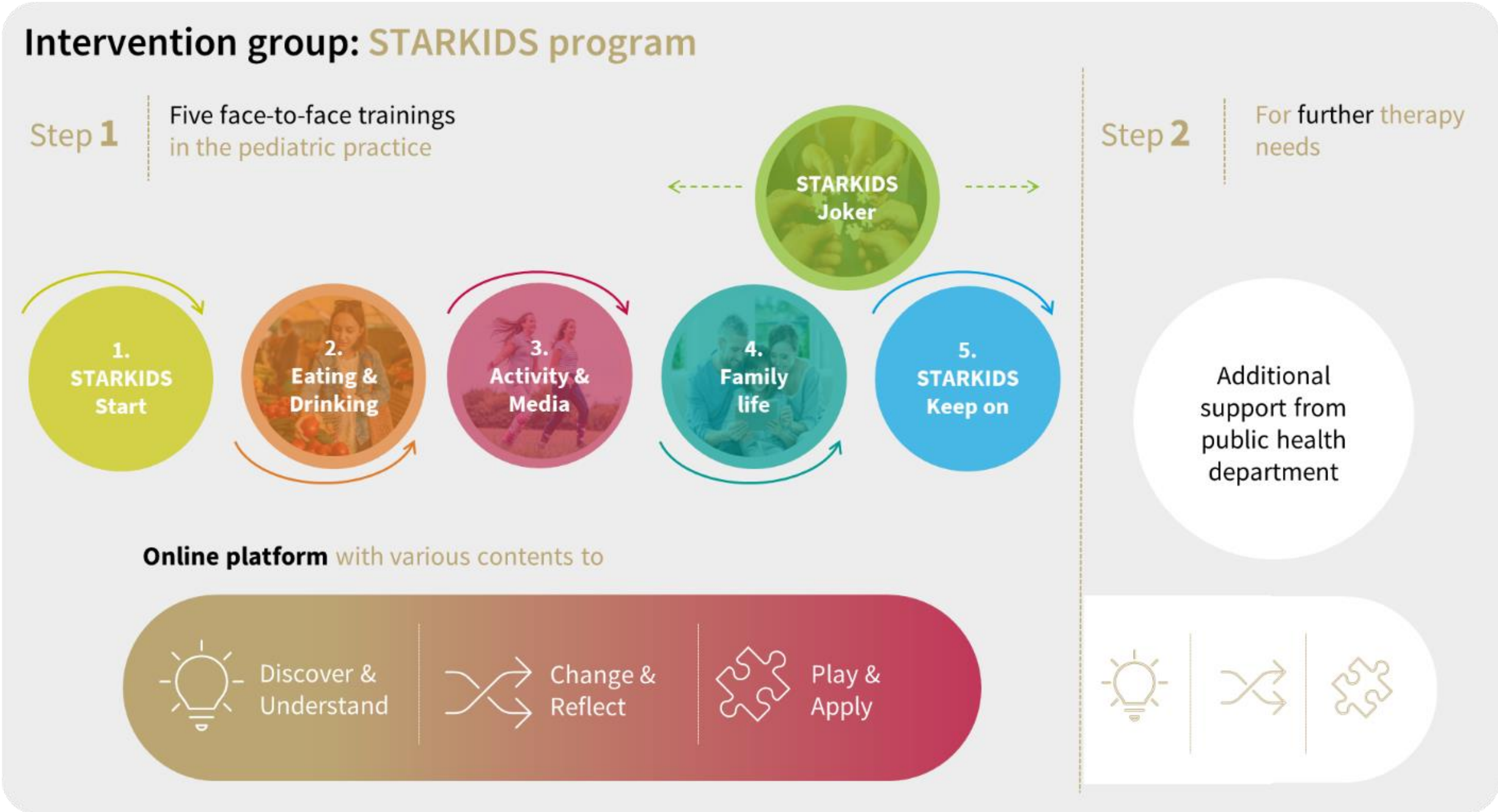

B: Overview face-to-face sessions

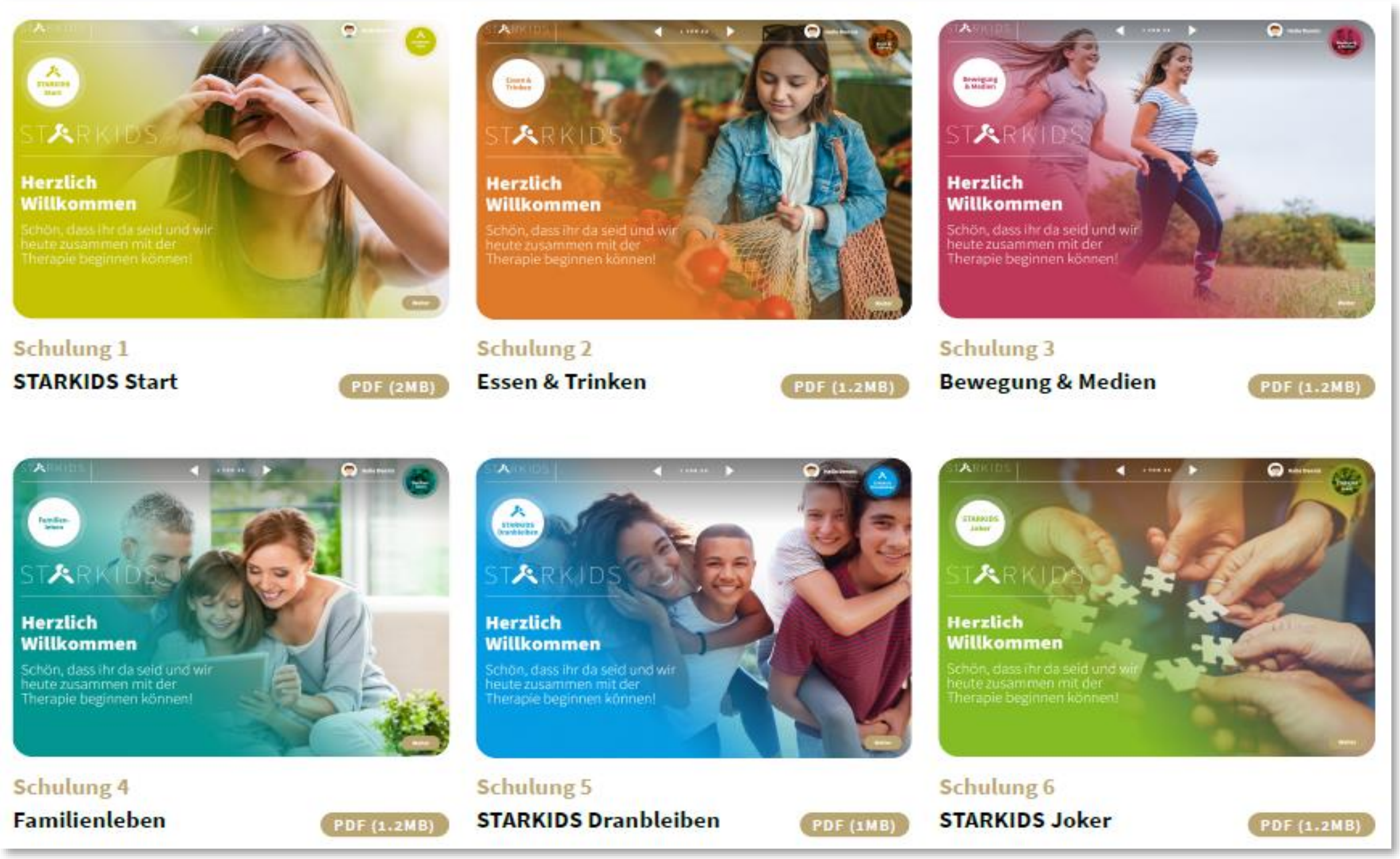

C: Overview online platform

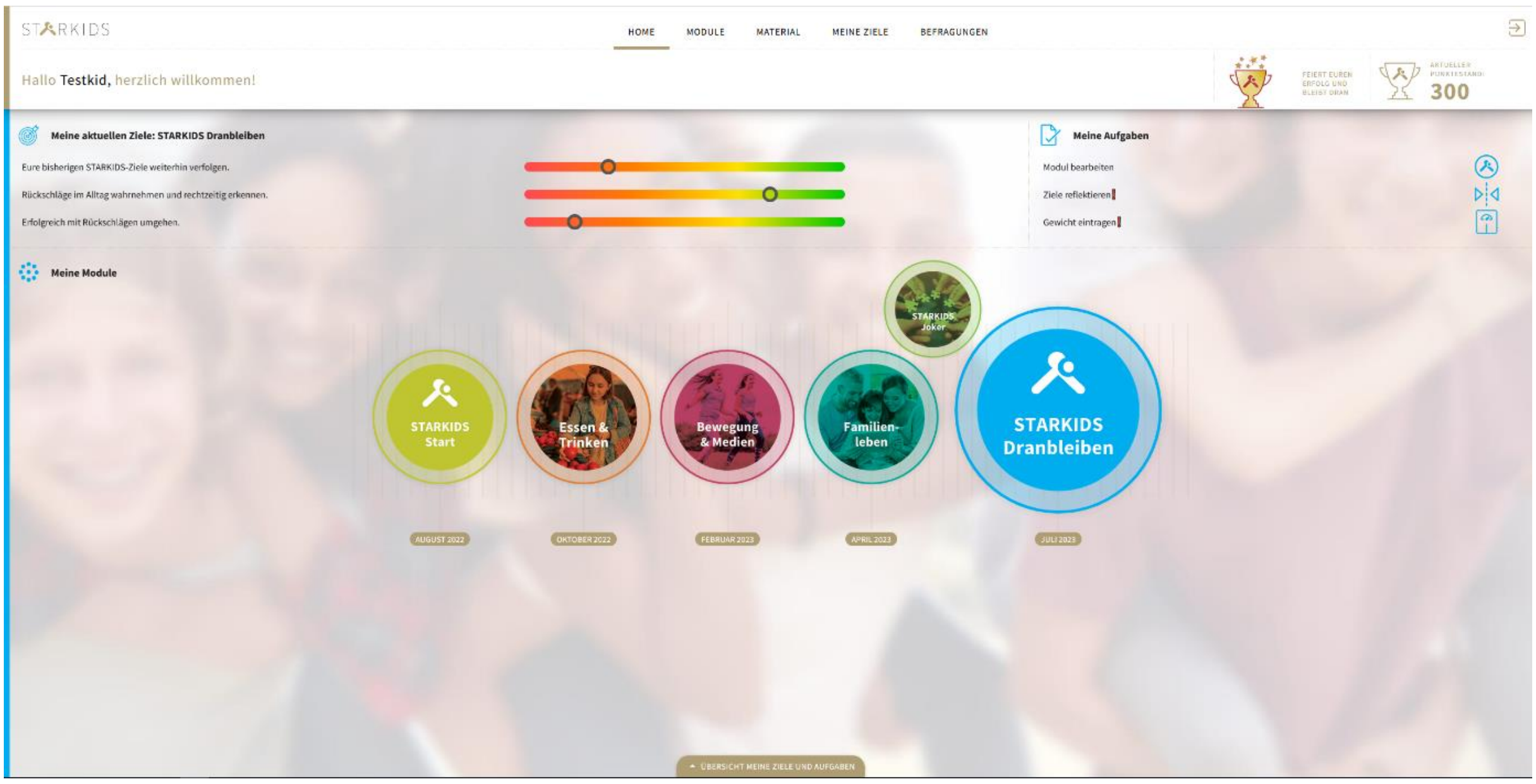

Notes. Online platform: Top left: STARKIDS aims that are reflected on every two weeks with indicators showing the last reflection. Top right: Tasks to complete in the program. Bottom center: All unlocked modules. By clicking on the module circle, families are forwarded to the contents of the specific modules containing the slides of the face-to-face sessions, informative texts, videos, serious games, reflection tools and other resources.

Supplementary figure 2: Weight class affiliation at the three measurement timepoints by group

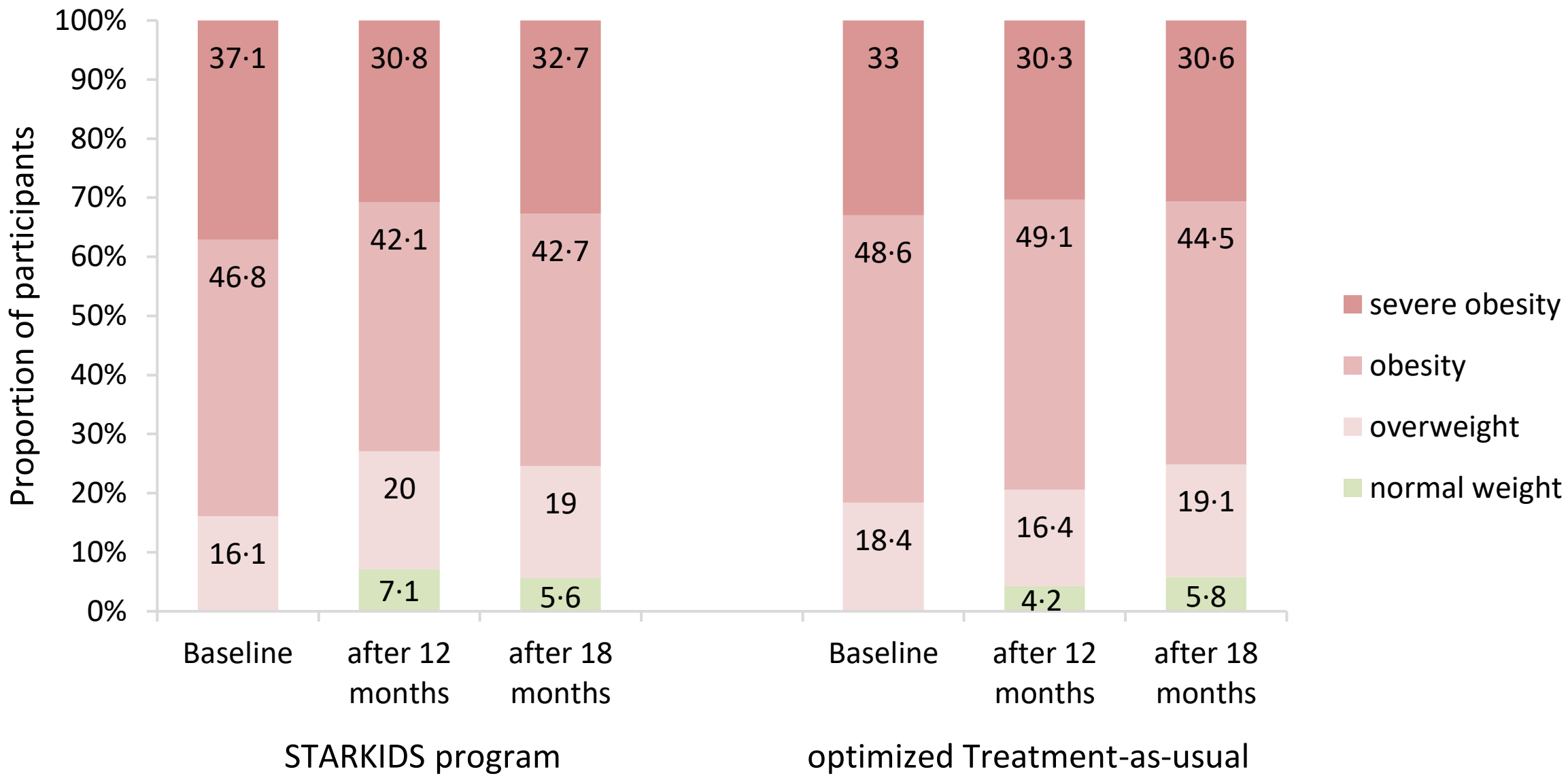

Supplementary figure 3: Proportion of participants with ≥ 0·2 BMI z-score reduction by group

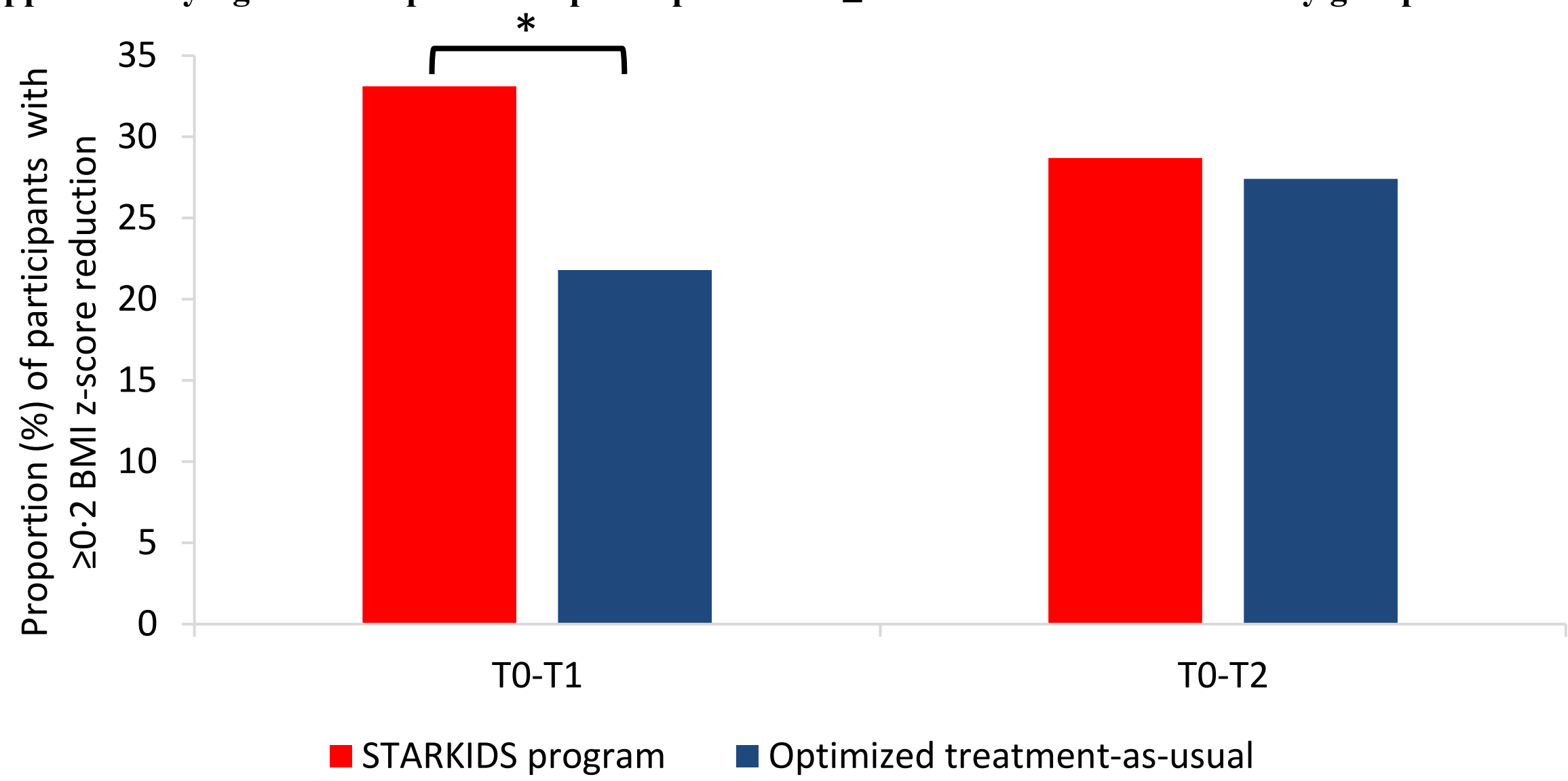

Supplementary table 1: Feasibility-related secondary outcomes reported in the current manuscript

Outcome domain	Outcome measure	Respondent	Assessment time point
Intervention uptake	Attendance rates of face-to-face sessions (step 1), uptake of further counselling at local public health department (step 2)	Platform log data	T0-T2
Online platform usage	Login frequency, proportion of on-users, platform engagement according to type	Platform log data	T0-T2
Quantitative process evaluation	Feasibility encompassed:<br>(1) motivation, enjoyment, and feasibility of delivering the program, rated by practice staff (e.g., "STARKIDS is feasible for implementation in pediatric practices").<br>(2) satisfaction with the STARKIDS program (face-to-face sessions, online platform, further counselling at local public health department), rated by parents/legal guardians and adolescents (e.g., "I am satisfied with STARKIDS.)"<br><br>All items were assessed on a 5-point Likert scale (1 = strongly disagree, 5 = strongly agree)	Practice staff, parents/legal guardians, adolescents	T1 and T2
Qualitative process evaluation (briefly referenced in the discussion, full reporting in a separate manuscript)	Semi-structured qualitative interviews of intervention experiences and barriers to uptake	subsample of parents/legal guardians and adolescents	between T1 and T2

Supplementary table 2: Coefficients for mixed models with BMI z-score as a continuous outcome variable

Term	Coefficient	SE	Wald	df	p
Expected value ('mean') CG, t0	2·37	0·04	67·44	1633·96	<·0001
Difference t0>t1, CG	-0·05	0·02	-1·95	1157·51	0·0515
Difference t0>t2, CG	-0·08	0·03	-2·94	1284·25	**0·0003**
Difference IG - CG, t0	0·05	0·04	1·04	1624·72	0·2964
Difference t0>t1 (IG) - difference t0>t1 (CG)	-0·09	0·03	-2·90	1120·21	**0·0038**
Difference t0>t2 (IG) - Difference t0>t2 (CG)	-0·03	0·04	-0·86	1175·75	0·3873

Notes. CG = control group, IG = intervention group

Supplementary table 3: Attendance rates and usage data of the STARKIDS program

**Face-to-face training sessions**	**Attendance in the STARKIDS program (n=343)**
STARKIDS Start	314 (92%)
Eating & Drinking	279 (81%)
Activity & Media	253 (74%)
Family Life	246 (72%)
STARKIDS Keep on	236 (69%)
STARKIDS Joker	10 (3%)
Counseling at the local health department (step 2)	24/162 (15%)

**Contents of the online platform according to type**	**Click frequencies of users M(SD), [range]**	**proportion of non-users (incl. dropouts)**
Webinars	12·19 (10·86), [1-75]	16%
Tips Paper	7·91 (9·58), [1-82]	32%
Videos	18·14 (20·64), [1-130]	36%
Reflection tools	11·46 (16·67), [1-117]	46%
Serious games	7·69 (8·55), [1-57]	53%
Mindfulness audios	2·52 (2·48), [1-12]	78%

Notes. M = mean, SD = standard deviation

Supplementary file 1: (Serious) Adverse Events

(Serious) Adverse Events
Adverse events (AEs) are distinguished from serious adverse events (SAEs). To enable documentation appropriate for healthcare research studies, the following (S)AEs are defined for STARKIDS and are subject to mandatory reporting:

Adverse Event (AE)
In general, adverse events are defined as any unfavourable medical occurrence in a patient, where the event does not necessarily have a causal relationship with the treatment.

The following AEs are specifically defined for STARKIDS:

- Persistent, serious, and marked medical complications — such as malignancies — that require medical treatment and did not exist as chronic conditions prior to the intervention;
- Persistent, serious, and marked psychological decompensation, i.e. newly occurring symptoms that did not exist as chronic conditions prior to the intervention, such as suicidality, self-harm, deterioration of mood, newly onset binge eating behaviour, or extreme avoidance behaviour (e.g. persistent inability to attend school; persistent incapacity to work with receipt of pension or insurance benefits);
- Persistent, serious, and marked interpersonal problems that did not exist prior to the intervention, such as social conflicts with family members or relationship separations.

Note: General weight changes and non-compliance (e.g. unexcused absence) are recorded directly in the online portal and are not listed separately as AEs.

If an AE occurs, the AE — including the reasons — is documented by the pediatric practice or the receiving facility (e.g. the coordinating centre) on the designated reporting form, and the study management is informed accordingly. **The treating pediatrician makes the decision regarding further clinical management and documents this on the reporting form as well.** A summary of AEs is reported to the Data Safety and Monitoring Board (DSMB) at regular intervals.

Serious Adverse Event (SAE)
Adverse events are generally classified as serious if they are:

- Life-threatening (e.g. acute suicidality);
- Fatal.

The following potentially life-threatening SAEs are specifically defined for STARKIDS:

- Acute medical complications requiring immediate inpatient treatment;
- Acute psychological decompensation with indication for immediate inpatient treatment, such as acute suicidality, severe self-harm, or severe depressive episode.

All SAEs are to be reported immediately to the central study management. Subsequently, the SAE is reported to the Ethics Committee of the Baden-Württemberg State Medical Association (Ethikkommission der Landesärztekammer Baden-Württemberg) and to the DSMB.
