# Supplementary material for: Effects of an e-health enhanced multicomponent outpatient intervention for children and adolescents with overweight or obesity in routine primary care (STARKIDS): a cluster-randomized controlled trial
Source: Lancet Reg Health Eur. 2026 Oct 14;71:101897. doi: 10.1016/j.lanepe.2026.101897 (PMC13694850; doi:10.1016/j.lanepe.2026.101897)
Supplement: Supplementary file 2 [file mmc3.pdf]

Study Protocol for the Scientific Investigation

Stepwise Model for Obesity Prevention and Treatment in Children and Adolescents (STARKIDS)

English translation of the protocol for submission to *The Lancet Regional Health - Europe*

Date: 26.03.2026 (based an the German version from 13.01.2021) **Version**: V1.2

Consortium Leadership

Prof. Dr. med. Florian Junne, M.Sc. IHP (LSE), Medical Director, Department of Psychosomatic Medicine and Psychotherapy, Otto von Guericke University Magdeburg, Leipziger Str. 44, 39120 Magdeburg, Tel.: 0391-67-14201, E-Mail: florian.junne@med.ovgu.de

Prof. Dr. med. Stefan Ehehalt, Public Health Department of Stuttgart, Schloßstr. 97, 71076 Stuttgart, Tel.: 0711-216-59430, E-Mail: stefan.ehehalt@stuttgart.de

Project Coordinator and Contact Person for the Ethics Committee

Dr. Katrin Ziser, University Hospital Tuebingen, Psychosomatic Medicine and Psychotherapy, Osianderstr. 5, 72076 Tuebingen, E-Mail: katrin.ziser@med.uni-tuebingen.de, Tel.: 07071 29-83610

Participating Researchers and Consortium Partners

University Hospital / University of Tuebingen
Prof. Dr. Peter Martus, Institute for Clinical Epidemiology and Applied Biometry
Prof. Dr. Isabelle Mack, Prof. Dr. Katrin Giel, Nutrition Science/Psychobiology
Prof. Dr. Andreas Nieß, Department of Sports Medicine
Prof. Dr. Ansgar Thiel, Department of Sports Science
Prof. Dr. Guido Zurstiege, Institute of Media Studies,
Prof. Dr. Wolfgang Bethge, Center for Clinical Trials

Central Institute of Mental Health Mannheim: Prof. Dr. Hans-Joachim Salize
Baden-Wuerttemberg State Health Office: Dr. Torben Sammet
Data Medico GmbH: Olaf Schliesing
AOK Baden-Wuerttemberg: Katrin Tomaschko

Cooperation partners

Professional Association of Pediatricians (BVKJ) in Baden-Wuerttemberg and BVKJ-Service GmbH, HÄVG AG, MEDIVERBUND AG, MEDI Baden-Wuerttemberg e.V.

Funding

Innovation Committee of the German Joint Federal Committee (G-BA), funding number 01NVF18013 (STARKIDS)

Content

1 Signature list and approval by the Medical Director ... 3
2 List of abbreviations ... 4
3 Study Description and Rationale ... 5
4 Study Objectives ... 6
5 Study Population ... 6
Recruitment ... 7
Sample Size Calculation ... 8
6 Study Duration ... 9
7 Study Procedures and Assessment Methods ... 10
7.1 Study Procedures ... 10
7.2 Quantitative Assessment Instruments ... 11
Primary Assessments ... 12
Lifestyle Assessment Instruments Relevant to Weight Development and Obesity-Related Comorbidities ... 12
Psychosocial Assessment Instruments ... 14
Health Economic Assessment Instruments ... 17
AOK Baden-Württemberg Routine Data ... 18
Process Evaluation (Quantitative) ... 18
7.3 Qualitative Instruments and Analyses ... 18
Qualitative Analysis of Family Mediation Styles and Responses to Advertising ... 18
Qualitative Analysis of Psychosocial Dimensions ... 19
Qualitative Analysis for Process Evaluation ... 19
Methodology of Qualitative Analyses ... 20
8 Risk/Benefit Assessment of Study Participation ... 20
9 Outcome Criteria ... 21
Primary Outcome Parameters ... 21
Secondary Outcome Parameters and Determinants of Treatment Course and Response 21
Statistical Analyses ... 22
10 Data Protection ... 22
11 Appendices ... 23
12 References ... 23

1 Signature list and approval by the Medical Director

Signatures

Place, Date	**Prof. Dr. med. Florian Junne, M.Sc.**<br>Medical Director<br>Department of Psychosomatic Medicine and Psychotherapy,<br>Otto von Guericke University Magdeburg
Place, Date	**Prof. Dr. med. Stefan Ehehalt**<br>Department Head<br>Public Health Department of Stuttgart
Place, Date	**Dr. Katrin Ziser**<br>Project Coordinator STARKIDS<br>Psychosomatic Medicine and Psychotherapy,<br>University Hospital Tuebingen

Approval by the Medical Director

I support the implementation of the project

„Stepwise Model for Obesity Prevention and Treatment in Children and Adolescents (STARKIDS)".

Place, Date	**Prof. Dr. med. Stephan Zipfel**<br>Medical Director<br>Psychosomatic Medicine and Psychotherapy,<br>University Hospital Tuebingen

2 List of abbreviations

AGA	Working Group on Childhood and Adolescent Obesity
BMI	Body Mass Index
BMI-SDS_{LMS}	Body Mass Index Standard Deviation Score (LMS-Method), BMI z-score
BMZI	Motives and Goals for Sport in Adolescents Questionnaire
BSA-F	Physical Activity and Sport Questionnaire
BVKJ	Professional Association of Pediatricians
ChEDE-Q8	Eating Disorder Examination - Questionnaire 8 for Children
DEBQ-K	Dutch Eating Behavior Questionnaire - Kinder
EDE-Q8	Eating Disorder Examination - Questionnaire
EDI-2	Eating Disorder Inventory - 2
FFQ	Food Frequency Questionnaire
HzV	Family Doctor program
ICD	International Classification of Diseases
IEG-K	Body Image in Children and Adolescents Questionnaire
KiGGS	Studie zur Gesundheit von Kindern und Jugendlichen in Deutschland
KINDL-R	Health-Related Quality of Life Questionnaire
KJAP	Pediatric Practice
MPA	Medical Assistant
MOMO-AFB	Physical Activity Questionnaire
MRV	Mannheim Module on Resource Consumption Questionnaire
PACES	Physical Activity Enjoyment Scale
PädNetzS e.G.	Cooperative for Specialized Medical Care for Children and Adolescents
PSQ-20	Perceived Stress Questionnaire
PURICA-S	Parent Perspective University of Rhode Island Change Assessment - Short
SD	Standard deviation
SDQ-Deu-S	Behavioural and Prosocial Functioning Questionnaire
SSK-Skala	Exercise- and Sport-Related Self-Concordance Scale
SU-F / SU-B	Sport-Related Social Support from Family and Acquaintances Questionnaire
URICA-S	University of Rhode Island Change Assessment - Short

3 Study Description and Rationale

Overweight and obesity represent one of the greatest current challenges facing healthcare systems (World Health Organization, 2021). In Germany, approximately two-thirds of men (67%) and half of women (53%) are currently overweight, and one quarter of adults (23% of men and 24% of women) are obese (Mensink et al., 2013). Among children and adolescents, approximately 15% are affected by overweight and 6% by obesity (Kurth & Rosario, 2007; Schienkiewitz, Brettschneider, Damerow, & Rosario, 2018).

Many of these children and adolescents remain obese and are exposed from an early age to an elevated risk of physical and mental comorbidities and sequelae, which are associated with reduced life expectancy and high (healthcare) costs (Effertz, Engel, Verheyen, & Linder, 2016; Giel et al., 2013; Linder et al., 2014). At the same time, guideline-compliant care structures and structured outpatient treatment concepts remain lacking, particularly for children and adolescents (Arbeitsgemeinschaft Adipositas im Kindes- und Jugendalter (AGA) & Deutsche Adipositas-Gesellschaft (DAG), 2019; Nolting, Krupka, Sydow, & Tisch, 2016). To close this gap in care, innovative, structured, and integrated services for the treatment of overweight and obesity in childhood and adolescence are urgently needed (Ebbeling, Pawlak, & Ludwig, 2002; Mühlig et al., 2017).

The aim of the present study is to evaluate a low-threshold, needs-based intervention for families with children and adolescents who are overweight or obese. Its contribution to the advancement of healthcare delivery consists of: (i) electronic collection of medical history and guideline-compliant diagnostics; (ii) a computer-assisted outpatient in-person training programme, conducted in the pediatric practice (KJAP) under physician supervision by the medical practice assistant (MPA) together with the affected families (Step 1); (iii) independent, cross-service case management by the public health service for overweight/obese children and adolescents with initially moderate or absent treatment response, for the purpose of further treatment planning (Step 2); (iv) an online resource for consolidating training content in the home environment, providing playful delivery of relevant content. A detailed description of the intervention is provided in Appendix 1.

The present evaluation study is conducted as a two-arm, prospective, cluster-randomised controlled trial (cRCT) with a control group design. The intervention group receives the newly developed intervention. In the control group, a single baseline counselling session ("treatment as usual", TAU) is provided at enrolment. Both groups undergo three primary assessment time points (T0, T1, T2).

The evaluation is based on established measures (e.g. BMI-SDS_{LMS}) as well as quantitative questionnaire-based measures (e.g. health-related quality of life and psycho-bio-social determinants) of treatment course and intervention outcome. Concurrent qualitative process evaluations are conducted (e.g. experiences of patients, MPAs, and pediatricians with individual intervention components, materials, and training sessions). All outcome parameters are clearly defined, measurable, and scientifically recognised variables, assessed using validated instruments.

4 Study Objectives

Immediate Objectives

(i) Reduction of the health burden attributable to overweight or obesity in participating children and adolescents;
(ii) Guideline-compliant diagnostics, early identification of overweight-related comorbidities, and guideline-compliant treatment;
(iii) A low-threshold, community-based, outpatient programme enabling comprehensive, needs-tailored, and differentiated care for families with affected children and adolescents;
(iv) Cost-effectiveness of the intervention compared to the control condition, with a positive cost–benefit value.

Intermediate Objectives

(i) Improved psychosocial well-being of families with affected children and adolescents, enhanced social participation, and reduced stigmatisation and exclusion;
(ii) A measurable and favourable Social Return on Investment.

Long-Term Effects (Impacts)

(i) Transferability of the concept to other regions of Germany;
(ii) Reduced mortality and morbidity in the affected population, and reduced morbidity-related costs to healthcare and social systems;
(iii) Intervention materials that remain usable and transferable in the long term.

5 Study Population

The study population consists of parents and their children and adolescents with overweight or obesity. For children and adolescents aged 3 to 11 years, only parents are surveyed using quantitative and qualitative methods. For children and adolescents aged 12 to 17 years, parents are likewise assessed quantitatively and qualitatively; in addition, the children and adolescents themselves are assessed, subject to prior parental consent. The inclusion and exclusion criteria for family participation are defined as follows:

Inclusion Criteria	Exclusion Criteria
At least one child or adolescent aged 3–17 years in the family with overweight (BMI > 90th percentile) or obesity (BMI > 97th percentile)	Prior participation in an interdisciplinary obesity training programme of at least one year's duration
Statutory health insurance coverage under the AOK Baden-Württemberg general practitioner-centred care contract (HzV contract, Pediatrics Module)	Participation in pediatric rehabilitation or inpatient intervention targeting weight loss within the past year

Adequate German language proficiency	Severe other systemic illness in the child or adolescent
	Severe other psychosocial burden or disorder in the child or adolescent not associated with overweight or obesity (e.g. schizophrenia spectrum disorder [ICD F2] in the context of treatment with appetite-stimulating medication [e.g. antipsychotics])

In accordance with Articles 19, 20, and 28 of the Declaration of Helsinki, the inclusion of minors is justified on the grounds that a group benefit for the participating minors can be assumed, alongside minimal risks associated with the study. To ensure the principle of minimal burden in the conduct of the study, children under the age of 11 years are not directly surveyed as part of the study (neither quantitatively nor qualitatively); they may, however, be included by their parents at their own discretion in the playful engagement with selected content. Children aged 12 years and above are assessed using an abbreviated version of the study questionnaires.

Recruitment

Recruitment of KJAPs is led by the consortium lead in close coordination with the BVKJ Service GmbH, the BVKJ e.V., MEDIVERBUND AG, MEDI Baden-Württemberg e.V., and AOK Baden-Württemberg, proceeding in a stepwise manner over the first 16 months of the project.

At the outset of the recruitment phase for N = 100 KJAPs, a STARKIDS website will already be available. The website will contain all information about the study project: advantages and benefits, aspects of relief and innovation, information on written, telephone, and in-person contact with the consortium lead, the option to request further written materials, and the possibility for pediatricians to register their own KJAP directly for participation in the project. The link to the STARKIDS website (https://starkids.medizin.uni-tuebingen.de; URL applied for) will be made available on the websites of all project partners, AOK, the State Health Office, and the Association of Pediatricians (BVKJ e.V.), accompanied by a brief description of the project. In addition, at the start of the recruitment phase, all potentially eligible KJAPs in Baden-Württemberg will be informed about the STARKIDS project (project objectives, participation requirements, remuneration, contact persons, etc.) by AOK Baden-Württemberg and BVKJ Service GmbH via electronic mail and postal distribution of flyers. Contact details for KJAPs may also be identified using the search function on the website of the Association of Statutory Health Insurance Physicians (https://www.arztsuche-bw.de/).

KJAPs wishing to participate in the project may register via two channels: either using the link provided in the email or via the STARKIDS website, or by contacting the consortium lead directly by telephone. In both cases, practices will be contacted by the consortium lead after registration for study briefing. At this point, any outstanding questions may be addressed and a training date for the practice team may be arranged.

In addition to direct contact with individual KJAPs, staff from the Department of Psychosomatic Medicine and Psychotherapy (consortium lead) will attend quality circles for pediatricians in Baden-Württemberg to present the STARKIDS project in person and address any questions

directly. As a further communication channel, BVKJ Service GmbH will report on the STARKIDS project at relevant events.

In addition, presentations of the project are planned in the *Ärzteblatt Baden-Württemberg* (http://www.aerzteblatt-bw.de/) and in the PädNetzS-Info newsletter of the specialist pediatric care cooperative (https://www.paednetzs.de/paednetzs-info/). Should all recruitment channels described above fail to yield the target of N = 100 KJAPs, all potentially eligible KJAPs will be contacted again by telephone by a member of the consortium lead team.

KJAPs participating in the project will subsequently receive flyers and informational materials for use in recruiting families with overweight or obese children and adolescents; these materials explain the STARKIDS project in a clear, lay-accessible manner and are designed to motivate families to participate.

Sample Size Calculation

The sample size calculation was conducted by Prof. Dr. Peter Martus (evaluating centre and consortium partner in the present project) as follows:

The statistical analysis addresses two primary endpoints: BMI-SDS and health-related quality of life, measured using the revised questionnaire for children and adolescents assessing health-related quality of life (KINDL-R) total score (Ravens-Sieberer, Ellert, & Erhart, 2007). A hierarchical testing procedure is applied, in which BMI is tested first as the more specific endpoint, with quality of life tested subsequently only if the result for BMI is statistically significant. Given this hierarchical approach, correction for multiplicity (e.g. Bonferroni) is not required. Analysis follows the intention-to-treat principle, with reporting of adherence, drop-out, and loss to follow-up (Mühlig, Wabitsch, Moss, & Hebebrand, 2014). With N = 2 × 500 patients (intervention and control groups) and an adherence rate of 75%, a null effect must be assumed for the remaining 25% of patients (primary refusers and drop-outs), resulting in a sample size inflation factor of 9/16. Furthermore, a cluster effect of 2 is assumed due to practice-based randomisation. This means that the recruitment of 2 × 500 patients, or 2 × 375 patients without drop-out, corresponds—after accounting for drop-out and the cluster effect—to an effective sample size of 2 × 140 patients (500 / (2 × 16/9)). The effective sample size is a technical term from the methodology of cluster-randomised trials, denoting the sample size that would yield the same statistical power in the absence of a cluster effect as the planned sample with a cluster effect. For this effective sample size, differences in the attainment of the primary endpoint (a reduction in BMI-SDS_{LMS} of ≥ 0.2, ranging from 14% [median proportion 20%] to 17% [median proportion 50%]) are detectable (chi-squared test, two-sided type I error of 5%, type II error of 20%, i.e. 80% power).

For the second primary endpoint (KINDL-R total score; Ravens-Sieberer et al., 2007), normative values stratified by age group, sex, and subscale have been published by the Robert Koch Institute. Back-calculation of confidence intervals to normative ranges is possible using the accompanying sample sizes. The standard deviation of the 0–100-scaled total score ranged from approximately 10–12 points across age and sex strata, with means between 73 and 81.

It is assumed that in a sample of overweight and obese children the variability will be approximately 1.4-fold greater (corresponding to twice the variance), yielding a standard deviation of 14–17 points. With an effective sample size of 2 × 140 patients and assuming this (interindividual) variability, differences of 6.3 points can be detected (two-sample t-test, two-sided type I error of 5%, type II error of 20%). Given that intraindividual variability is expected to be lower than interindividual variability, it is anticipated that differences on the order of 5 points should be detectable. This difference may be considered clinically meaningful on a scale of 0–100.

Among the secondary parameters of particular interest, GOT/GPT values within the normal range at the Central Laboratory of Tübingen University Hospital are defined as 0–39 U/l for children aged 10–16 years. A rough estimate (SD = range/4) yields a standard deviation of approximately 10 points; for younger children, approximately 15. Assuming a 1.4-fold increase in variability, standard deviations of approximately 14–21 U/l are obtained. Under analogous calculations, differences of 6–8 U/l can be detected; overall—assuming reduced intraindividual variability—differences of 5 U/l are detectable. Analogous calculations indicate that differences of approximately 15 units in total cholesterol are detectable.

Additional secondary parameters include questionnaire measures of general physical activity (Motorik-Modul Activity Questionnaire [MoMo-AFB]; Jekauc, Wagner, Kahlert, & Woll, 2013) and eating behaviour (Dutch Eating Behavior Questionnaire for Children [DEBQ-K]; Franzen & Florin, 1997). For the MoMo scale, standard deviations of 1.1 and 0.6 have been reported for children aged 9–12 and 14–17 years, respectively, with means of 11.8 and 14.5. Accordingly, differences of 0.2–0.4 points are detectable in the present study.

6 Study Duration

The total project duration is four years and encompasses intervention development, piloting, and KJAP recruitment (21 months), conduct of the evaluation study (24 months), and a period for data preparation and analysis (3 months). The official project start date was 15 September 2019.

The study duration and time burden for individual participants are estimated as follows:

Intervention Group Participants

- Study briefing and informed consent by the respective pediatrician (approx. 0.5 h); appointment scheduling with the supervising MPA (approx. 0.5 h in total)
- Participation in medical examinations at three primary assessment time points (approx. 0.5 h each)
- Participation in in-person training sessions at the KJAP, with a brief pre-session questionnaire (5–6 sessions of approx. 1.5 h each)
- Participation in questionnaire assessments at three primary time points (approx. 1 h each)

- If applicable, participation in case management at the relevant public health office (1–2 appointments of approx. 1 h each; only in cases of moderate or insufficient treatment response following Step 1)
- Use of the online platform as a supplement to the in-person sessions, at participants' own discretion
- Optional: participation in the qualitative process evaluation (approx. 1 h)

Control Group Participants

- Study briefing and informed consent by the respective pediatrician (approx. 0.5 h)
- Participation in medical examinations at three primary assessment time points (approx. 0.5 h each)
- Participation in a single baseline counselling session ("treatment as usual", approx. 0.5 h)
- Participation in questionnaire assessments at three primary time points (approx. 1 h each)
- Optional: participation in the qualitative process evaluation (approx. 1 h)

Pediatric Practices

- Participation in the intervention training workshop (approx. 4 h)
- Study briefing and obtaining informed consent (approx. 0.5 h per patient), appointment scheduling as required (approx. 0.5 h in total)
- Conduct of medical examinations at three primary assessment time points (approx. 0.5 h each)
- If an intervention practice: conduct of in-person training sessions (5–6 sessions of approx. 1.5 h each)
- Participation in quantitative process evaluation (approx. 1 h, distributed across various brief surveys)

Participating Public Health Offices (Step 2 of the Intervention)

- Participation in the Step 2 intervention training workshop (approx. 2 h)
- Conduct of case management/counselling for study participants with moderate or insufficient treatment response (1–2 appointments of approx. 1 h each)
- Documentation and brief survey (approx. 0.5 h)

7 Study Procedures and Assessment Methods

7.1 Study Procedures

Following completion of intervention development and piloting, participating KJAPs will be recruited (see Section 5, Study Population/Recruitment) and cluster-randomised. Both intervention and control practices will receive training, including all required materials and

equipment for their respective study condition. Following successful training, KJAPs will begin recruiting study participants ("screening").

Screening for overweight and obesity in participating KJAPs will occur in the context of routine practice visits (well-child examinations ["U-Untersuchungen"]) as well as through proactive identification and retrieval of previously documented BMI percentile values (> 90th percentile) via a query in the practice software, followed by study briefing and invitation to participate. In families with multiple overweight or obese children or adolescents, one child will be selected at random and invited to participate.

Families will be briefed about the study by the responsible pediatrician (including age-appropriate briefing of participating children and adolescents). Upon provision of informed consent, all practices will conduct assessment time point T0, comprising a medical examination including guideline-compliant laboratory investigations, a questionnaire assessment, and—depending on study condition—either a single baseline counselling session on overweight and obesity or the scheduling and conduct of the first in-person training session. In the intervention condition, the in-person training sessions are distributed over the course of one year. During this period, participating families will have access to supplementary online materials (e.g. webinars, educational videos, and learning games).

Twelve months after study enrolment, the second primary assessment time point (T1) will be conducted, comprising a medical examination including guideline-compliant laboratory investigations and a questionnaire assessment. In the intervention group, a determination will be made as to whether moderate or insufficient treatment response following Step 1 of the intervention has occurred (see Section 7.2, BMI-SDS_{LMS}). If this is the case, patients will be referred to the nearest participating public health office for further case management. At this time point, patients will also be informed by the study team about the qualitative process evaluation and approached regarding optional participation (see separate participant information sheet and informed consent form).

Eighteen months after study enrolment, the third primary assessment time point (T2) will be conducted, comprising a medical examination including guideline-compliant laboratory investigations and a questionnaire assessment; this time point simultaneously marks the end of the study. Patients will again, where applicable, be informed by the study team about the qualitative process evaluation and approached regarding optional participation (see separate participant information sheet and informed consent form).

7.2 Quantitative Assessment Instruments

At each of the three primary assessment time points T0, T1, and T2, the two primary outcome parameters—BMI and quality of life (see Section 9, Outcome Criteria)—as well as secondary outcome parameters and determinants of treatment course are assessed, with a focus on the psycho-bio-social dimensions of the intervention, using questionnaire-based instruments.

Primary Assessments

BMI-SDS$_{LMS}$

Body mass index (BMI; body weight / body height² [kg/m²]) is calculated from the height and weight of the children and adolescents, and BMI percentile is derived with reference to age and sex. Overweight is defined as a BMI above the 90th percentile; obesity as a BMI above the 97th percentile. Percentile curves are defined in accordance with the recommendations of the Working Group on Obesity in Childhood and Adolescence (K. Kromeyer-Hauschild, Moss, & Wabitsch, 2015; Katrin Kromeyer-Hauschild et al., 2001). To evaluate treatment response, the BMI-SDS$_{LMS}$ is calculated, which is standardised for age and sex and thereby enables a uniform cut-off for all children and adolescents. Following Step 1 of the intervention, patients are categorised according to their BMI-SDS$_{LMS}$ for Step 2: a BMI reduction of ≥ 0.2 SDS$_{LMS}$ constitutes treatment success; a BMI reduction of 0 to < 0.2 SDS$_{LMS}$ constitutes moderate treatment response; and an increase in BMI-SDS$_{LMS}$ constitutes treatment failure.

Health-Related Quality of Life (KINDL-R Questionnaire)

The KINDL-R is a German-language questionnaire instrument for the assessment of subjective health-related quality of life in children and adolescents. Both the self-report and parent-report versions of the KINDL-R can be administered for individuals aged 3–17 years in both healthy and clinical populations. Health-related quality of life (total score) is assessed across six dimensions (physical well-being, emotional well-being, self-esteem, family well-being, peer/social well-being, school well-being) using 24 items rated on a five-point Likert scale (never, seldom, sometimes, often, always). Scores are expressed on a 0–100 scale, with higher values indicating greater quality of life. In the German Health Interview and Examination Survey for Children and Adolescents (KiGGS; Hölling et al., 2012), very good to excellent internal consistency was observed for almost all subscales and the total score (Cronbach's α = .70–.86). Factorial validity of the questionnaire has also been confirmed (Ravens-Sieberer et al., 2007). In addition to the general KINDL-R, the Obesity Module (15 items) is employed to assess disease-specific quality of life.

Lifestyle Assessment Instruments Relevant to Weight Development and Obesity-Related Comorbidities

Eating Behaviour (DEBQ-K)

Restrained eating behaviour in children and adolescents is assessed using the DEBQ-K (Franzen & Florin, 1997). This self-report questionnaire for children and adolescents aged 9–15 years assesses three dimensions of eating behaviour—restrained eating, emotional eating, and external eating—across 30 items rated on a four-point Likert scale. All three scales demonstrate good to excellent internal consistency (α = .79–.95). With respect to criterion validity, high positive correlations (r = .74–.85) have been found between the restrained eating subscale of the DEBQ-K and the corresponding subscale of the Fear of Weight Gain Questionnaire (FAD; Franzen & Florin, 1997).

Physical Activity (MoMo-AFB)

Physical activity in children and adolescents is assessed using the MoMo-AFB (Jekauc, Voelkle, Wagner, Mewes, & Woll, 2013). The questionnaire comprises 28 items, of which 2 items assess general physical activity, while the remaining 26 items capture physical activity in daily life, in school, in organised leisure sport, and in unorganised leisure sport. For each of the four domains, the type, duration, frequency, and intensity of physical activity are recorded. Good to very good test–retest reliability has been demonstrated for children aged 9–17 years, with a mean kappa coefficient of .66 (SD = .19) across all items considered. Regarding criterion validity, correlations of r = .29 with accelerometer data have been observed, which are comparable to other physical activity questionnaires for children and adolescents (Jekauc, Wagner, et al., 2013).

Exercise- and Sport-Related Self-Concordance (SSK Scale)

The SSK Scale by Seelig and Fuchs (2006) is a self-report instrument assessing the concept of self-concordance (Deci & Ryan, 2000) across 12 items. It is a German-language, economical, valid, and reliable measure for adults. Self-concordance is conceptualised as a component of goal intention, reflecting the degree to which the goal intention is consistent with personal values and interests (ego-proximity). The SSK Scale assesses the exercise-related ego-proximity of goal intentions across four subscales (intrinsic, identified, introjected, and extrinsic motivational modes); participants rate their reasons for engaging in physical activity on a six-point Likert scale. In addition to the subscales, an overall self-concordance score can be computed (Seelig & Fuchs, 2006).

Media Use and Sleep

A central component of the construct of media literacy is the capacity to protect oneself from persuasive food marketing strategies, as well as to actively seek health-relevant information—for example, in the context of the planned intervention. A dual assessment of media literacy is therefore pursued:

- (i) In the broader sense of *media health literacy* (Levin-Zamir, Lemish, & Gofin, 2011), assessing both the capacity for active information-seeking and the capacity for self-protection in the context of potentially harmful content.
- (ii) (ii) In the narrow sense of competence in recognising the hazardous potential of marketing for a product category particularly relevant to overweight—namely, sugar-sweetened beverages (Chen, Porter, Estabrooks, & Zoellner, 2017).

In addition, screen use items from KiGGS Wave 1 (Manz et al., 2014) are used to assess media use, and items from the KiGGS baseline survey on average sleep duration and problems with sleep onset and maintenance are used to assess sleep (Schlarb, Gulewitsch, Weltzer, Ellert, & Enck, 2015).

<u>Psychosocial Assessment Instruments</u>

Readiness to Change (PURICA-S; URICA-S)

The Parent Perspective University of Rhode Island Change Assessment – Short (PURICA-S; Junne et al., 2019) is an instrument for assessing parental readiness to change regarding a problem area of their child, based on the established self-perspective measure of readiness to change URICA-S (University of Rhode Island Change Assessment - Short; Mander et al., 2012). The assessment comprises 16 items corresponding to four scales representing the stages of motivational change (precontemplation, contemplation, action, maintenance), each rated on a five-point Likert scale (0 = "not at all applicable", 4 = "very strongly applicable"). Validation was conducted in a sample of parents of overweight or obese children (Junne et al., 2019), yielding high internal consistency for three subscales (all α's = 0.8), and good validity. The precontemplation subscale did not achieve adequate internal consistency in the validation sample due to a floor effect.

Eating Disorder Psychopathology in Children and Adolescents (ChEDE-Q8)

Eating disorder psychopathology in children and adolescents is assessed using the German version of the Eating Disorder Examination–Questionnaire 8 for Children (ChEDE-Q8; Kliem et al., 2017). This self-report instrument for children and adolescents enables assessment of eating disorder symptomatology over the past 28 days on a seven-point Likert scale (0 = "no day", 6 = "every day") across four subscales (dietary restraint, eating concern, weight concern, and shape concern), as well as computation of a global score. The validation study by Kliem et al. (2017) demonstrated high internal consistency (α = 0.8) and satisfactory convergent validity (e.g. correlation of r = 0.37 with the Emotional Problems subscale of the Strengths and Difficulties Questionnaire) in children and adolescents aged 7–18 years.

Eating Disorder Psychopathology in Adults (EDE-Q8)

Eating disorder psychopathology in parents is assessed using the German version of the Eating Disorder Examination–Questionnaire 8 (EDE-Q8; Kliem et al., 2016). This eight-item short form comprises two items per subscale across the four dimensions of dietary restraint, eating concern, weight concern, and shape concern, rated on a seven-point Likert scale (0 = "no day", 6 = "every day"). Excellent internal consistency (α = 0.9) and good construct validity have been demonstrated for this short version (correlation of r = 0.75 with the EAT-13; Berger et al., 2012).

Family Functioning (SCORE-15)

Perceived family functioning is assessed using the German version of the SCORE-15 (https://www.aft.org.uk/view/15101224f1e.html), a 15-item short form rated on a five-point Likert scale (1 = "strongly agree", 5 = "strongly disagree"). A total score can be computed, with higher values indicating a more negative appraisal of the family. Subscale scores can additionally be computed for three dimensions: Strengths and Adaptability, Overwhelmed by Difficulties, and Disrupted Communication. Validation of the English-language version

(Stratton et al., 2014) in adolescents and adults aged 11–84 years demonstrated good internal consistency (α = 0.89).

Perceived Stress (PSQ-20)

Subjective perception, appraisal, and coping with stressors over the preceding four weeks is assessed in participating parents using the German version of the Perceived Stress Questionnaire–20 (PSQ-20; Fliege, Rose, Arck, Levenstein, & Klapp, 2001). Five items each correspond to four subscales—Worries, Tension, Joy, and Demands—rated on a four-point Likert scale (1 = "almost never", 4 = "most of the time"), from which both subscale scores and a total score can be derived. Higher scores indicate greater expression of the respective construct. The validation study by Fliege, Rose, Arck, Levenstein, and Klapp (2001) demonstrated very good internal consistency of the subscales (α = .80–.86) and very good split-half reliability of the total score (r [Guttman] = .86). With respect to validity, a correlation of r = −.79 was found between the PSQ-20 total score and the psychological domain of the WHOQOL-Bref quality of life questionnaire (Angermeyer, Kilian, & Matschinger, 2000).

Family Climate and Shared Activities

Family climate is assessed using a subscale of the adolescent questionnaire from the iFamily study (Bammann, Reisch, Pohlabeln, Williams, & Wolters, 2019). Block 8, comprising 8 items rated on a five-point Likert scale (0 = "not at all applicable", 4 = "very strongly applicable"), asks respondents to indicate the extent to which certain adjectives describe the family atmosphere. Items are grouped into three subscales, with scores reflecting whether values equal or exceed the median, indicating higher perception of the emotional atmosphere. The three scales refer to perceived warmth (4 items, Cronbach's α = .83), strictness (1 item), and perceived tension in the relationship (3 items, Cronbach's α = .61; Elsayed et al., 2020; Latendresse et al., 2009). In addition, Block 9, comprising six items on shared family activities from the iFamily study (Bammann et al., 2019), is used. A five-point Likert scale (0 = "never", 4 = "every day") is used to assess how frequently activities such as sport or cultural pursuits are engaged in together with parents.

Behavioural and Prosocial Functioning (SDQ-Deu-S)

Behavioural difficulties and prosocial behaviour are assessed using the German self-report version of the Strengths and Difficulties Questionnaire (SDQ-Deu-S; Lohbeck, Schultheiß, Petermann, & Petermann, 2015). The questionnaire is available for children and adolescents aged 2–17 years in a parent-report version and a self-report version for children aged 11 years and above. The 25 items are distributed across five scales: emotional problems, externalising behavioural difficulties, hyperactivity/inattention problems, peer problems, and prosocial behaviour. The scales assessing behavioural difficulties can be summed into a total difficulties score, with classifications of normal, borderline, and abnormal. Psychometric studies have reported heterogeneous reliability findings, particularly with respect to test–retest reliability for the self-report version in children aged 11–16 years (r = .58–.67).

Body Image in Children and Adolescents (IEG-K "Body Dissatisfaction" Subscale)

Body image in children and adolescents is assessed using the "Body Dissatisfaction" subscale of the Inventory of Eating Behaviour and Weight Problems in Children (IEG-Kind), validated for children and adolescents aged 11–16 years (Diehl, 1999). The subscale comprises five items rated from 0 (does not apply at all) to 3 (fully applies), with excellent internal consistency ($\alpha = .88$).

Body Image in Adults (EDI-2 "Body Dissatisfaction" Subscale)

Body image in adults (parents) is assessed using the "Body Dissatisfaction" subscale of the German version of the Eating Disorder Inventory–2 (EDI-2; Paul & Thiel, 2005). This subscale comprises 9 items rated on a six-point Likert scale (1 = "never", 6 = "always"). Internal consistency in the normative sample was excellent ($\alpha = 0.91$; Salbach-Andrae et al., 2010). Regarding convergent validity, a correlation of $r = 0.52$ was found between the Body Dissatisfaction subscale and the Weight Phobia item from the SIAB-EX clinical interview for eating disorder symptoms (Fichter & Quadflieg, 1999).

Physical Activity and Sport Questionnaire (BSA-F)

Physical activity and sport behaviour in the parents of affected children and adolescents is assessed using the Physical Activity and Sport Questionnaire (BSA-F; Fuchs, Klaperski, Gerber, & Seelig, 2015), comprising three sections measuring occupational physical activity, leisure-time physical activity, and sport activity. The questionnaire is based on the FITT framework, assessing the frequency, intensity, duration, and type of physical activity across 15 items in various response formats. Validity was established through correlation with cycle ergometer parameters of aerobic capacity, yielding correlations of $r = .32$–$.34$ with the BSA-F total activity index (Fuchs et al., 2015).

Physical Activity Enjoyment (PACES)

Enjoyment of physical activity is assessed using the German version of the Physical Activity Enjoyment Scale for children and adolescents (PACES; Jekauc, Voelkle, Wagner, Mewes, & Woll, 2013), comprising 16 items rated on a five-point Likert scale (1 = "strongly disagree", 5 = "strongly agree"). In a validation study with German children, Jekauc and colleagues (2013) demonstrated good to very good test–retest reliability ($r = .89$–$.93$), predictive validity of $r = .42$ (correlation with the PDPAR; Trost, 2007), and a correlation of $r = .16$ with accelerometry.

Motives and Goals for Sport in Adolescents (BMZI)

Sport-related motives and goals in adolescents are assessed using the Berne Motives and Goals Inventory for Adolescents (BMZI; Gut, Schmid, Schmid, & Conzelmann, 2019), which assesses 8 goals: social contact, competition/performance, distraction/catharsis, body/appearance, health, fitness, aesthetics, and risk/challenge. This self-report questionnaire comprises 26 items rated on a five-point Likert scale (1 = "does not apply", 5 = "applies"). Good reliability (test–retest reliability: $r = .62$–$.83$) has been demonstrated, and criterion validity

shows small to moderate correlations with the corresponding motivational modes of the SSK Scale (Seelig & Fuchs, 2006), with the exception of the social contact goal.

Dietary Assessment (Food Frequency Questionnaire)

Habitual dietary behaviour is assessed using a German version of the Food Frequency Questionnaire (FFQ; Mensink & Burger, 2004), originally developed for the KiGGS study (Hölling et al., 2012). Two versions are available, differing in phrasing: a self-report version (for children and adolescents aged 12 years and above) and a parent-report version. The questionnaire assesses the frequency and quantity of consumption of 45 food items. In a validation study (Truthmann, Mensink, & Richter, 2011), correlations of $r = .22–.69$ were found with the DISHES dietary interview (Mensink, Haftenberger, & Thamm, 2001), with the exception of pasta and rice.

Sport-Related Social Support from Family (SU-F) and Acquaintances (SU-B)

Two scales assess distinct sources of social support for engaging in sport: family and acquaintances (Fuchs, 1997). Items assess the degree to which these sources encourage, prompt, remind, assist, or jointly engage in physical activity. The family support scale comprises six items; the acquaintances support scale comprises five items. Both are rated on a four-point Likert scale (1 = "(almost) never", 4 = "(almost) always") and demonstrate very good reliability (Cronbach's $\alpha = .85$ for family; .89 for acquaintances).

<u>Health Economic Assessment Instruments</u>

Direct Intervention Costs

Direct intervention costs in both the intervention and control groups are recorded and used to calculate cost-effectiveness. The effectiveness criterion is the clinical endpoint at time point T2 described above: a reduction in $\text{BMI-SDS}_{\text{LMS}}$ of ≥ 0.2 (point prevalence).

Healthcare Utilisation Costs

Costs arising from utilisation of medical, psychosocial, and other study-relevant services by study participants are assessed longitudinally. At each of the three assessment time points, the frequency and duration of use of specific services are retrospectively quantified. The established "Mannheim Module on Resource Consumption" (MRV; Salize & Kilian, 2010), adapted for the specific study population, is used for this purpose.

Costs per Quality-Adjusted Life Year

Changes in longitudinally assessed KINDL total scores (health-related quality of life, see above) are used to generate preference-based utility values (QALYs). These are used to calculate the costs of quality-adjusted life years gained through the study interventions.

AOK Baden-Württemberg Routine Data

To conduct the health economic evaluation of the intervention (see Section 9, Outcome Criteria), statutory health insurance routine data from AOK Baden-Württemberg are retrieved for study participants. These encompass the following categories: demographic master data, outpatient care, inpatient care, pharmaceuticals, therapeutic and assistive devices, medical rehabilitation, preventive services and health promotion, medical treatments in healthcare facilities, and disease management programmes (DMPs). A separate application for authorisation under § 75 SGB X (Book X of the German Social Code) to retrieve routine data has been jointly initiated with AOK Baden-Württemberg.

Process Evaluation (Quantitative)

For quantitative process evaluation, various brief surveys are conducted with patients and participating practices to assess the in-person training sessions and online materials, particularly regarding their appropriateness (session frequency, quantity of materials, scope for individual adaptation) and utility (Were certain materials particularly helpful or unhelpful? What should be modified from the user perspective?), presented using Likert scales and, in part, free-text response formats.

In addition, usage data from the online platform are collected to supplement subjective patient-reported assessments, enabling inference regarding which content is used extensively and frequently, and which is used rarely or not at all.

7.3 Qualitative Instruments and Analyses

In addition to quantitative assessments, the STARKIDS project conducts non-participant observations, semi-structured interviews, and focus groups addressing family mediation styles and responses to advertising, psychosocial dimensions, and process evaluation of the intervention.

Qualitative Analysis of Family Mediation Styles and Responses to Advertising

The focus is on media-related influences on children's physical health. The qualitative analysis is designed to address how parents and children discuss harmful media influences with one another and negotiate the boundaries of children's media use in this context. This parent–child interaction is examined using a qualitative approach. Participants are provided with materials covering various forms of advertising directed at children, designed to stimulate, structure, and focus advertising-related conversations between parents and children on relevant aspects of these advertising forms.

Approximately 30 conversations between one parent and one of their children in the 8–12 age group are planned. The conversations are audio-recorded by the participating families using a provided dictation device, which is returned to the study team upon completion of data collection. The data thus collected are transcribed and analysed using content analysis.

The structured, playfully oriented task is expected to take approximately 30–45 minutes and is conducted independently by the parent together with the child in the family home. As all materials are sent by post, the physical presence of the study team during the conversation is not required.

Qualitative Analysis of Psychosocial Dimensions

In addition to media use, children and parents are also interviewed on psychosocial dimensions through semi-structured interviews and focus groups. Topics such as how overweight is addressed and communicated within the family, and barriers to healthy weight development in everyday life, represent key areas of barriers and adherence research. The following research questions, among others, are posed to children and parents: What makes it difficult to support healthy weight development in your child's daily life? If your child were of normal weight, what would be different in your daily family life compared to now? When talking with your child about their weight development, have there been or are there any difficult conversations? What motivates your family in pursuing your child's healthy weight development?

Individual interviews and focus groups addressing psychosocial dimensions will be conducted in summer 2020 at two sites (Stuttgart Public Health Office and Wangen Specialised Clinic) either by telephone or in person. During these interviews and focus groups, participants will also be presented with a preliminary version of the online resource and the in-person training programme. As these qualitative interviews are expected to raise thematic awareness in participants, they will be conducted with non-participating families. A total of 1 focus group with adolescents aged over 12 years (N = 10), 1 focus group with parents (N = 10), individual interviews with adolescents aged over 12 years (N = 5), parents (N = 5), and staff (N = 5) are planned.

Qualitative Analysis for Process Evaluation

As part of a target-group-focused process evaluation, the experiences, perspectives, and suggestions for improvement regarding the STARKIDS intervention will be collected prior to the start of the intervention from adolescents aged over 12 years (N = 10), parents (N = 10), and staff (N = 10) at a specialised clinic in Wangen using the "Speak Out Loud" method, followed by separate focus groups with adolescents, parents, and staff to discuss findings, in autumn 2020. As qualitative interviews are expected to raise thematic awareness in participants, these will likewise be conducted with non-participating families.

In addition, adolescents aged over 12 years (N = 20) and parents (N = 40; of whom N = 20 are parents of children under 12 years and N = 20 are parents of adolescents over 12 years) from the intervention group will be interviewed individually face-to-face or by telephone regarding their experiences, perspectives, and suggestions for improvement upon completion of Step 1 of the intervention. Following completion of Step 2 of the intervention, adolescents aged over 12 years (N = 10) and parents (N = 20; of whom N = 10 are parents of children under 12 years and N = 10 are parents of adolescents over 12 years) from the intervention group, as well as participants from the control group (N = 10 adolescents and parents), will again be interviewed individually face-to-face or by telephone to inform the adaptation and optimisation of the intervention, with a view to generating insights for potential transfer into routine care.

Methodology of Qualitative Analyses

Interview guides for the semi-structured interviews and focus groups addressing psychosocial dimensions and process evaluation are developed using the SPSS procedure (Collecting, Checking, Structuring, Subsuming) through a discursive process within the study group (see Appendix 9 for current status of guide development). Individual interviews and focus groups are audio-recorded during the sessions to allow for external transcription of qualitative material.

During transcription, all transcripts are fully anonymised so that no inferences can be drawn regarding participating individuals or institutions. All data protection requirements are adhered to and rigorously monitored. Participants or their legal guardians must provide written consent—by signing the informed consent form—prior to participation in and analysis of data from the qualitative assessments. Prior to this, participants and, where applicable, their legal guardians are informed about the nature and scope of the interviews via a participant information sheet (informed consent form and participant information sheet are provided in the appendix).

Content analysis of the transcribed written material is conducted following Mayring (2010). Category formation for individual interviews is primarily inductive, supplemented deductively in accordance with the guiding research questions. Coding is initially conducted by two independent coders through a process of communicative validation, during which both the categories and coding rules are iteratively refined. Subsequent coding and analysis are conducted using MAXQDA software. In an additional feedback loop, randomly selected transcripts coded by individual analysts are reviewed and discussed within the study group as a quality check. Findings are then summarised and reported in written form.

In accordance with standard regulations, individual interview and focus group transcripts are stored for ten years on secured data storage media in locked cabinets at the Department of Psychosomatic Medicine and Psychotherapy. Audio recordings of focus groups are deleted immediately after transcription to written form (generally within eight weeks of recording). After the ten-year period, all data are destroyed in accordance with applicable data protection regulations. The strictly confidential handling of all data complies fully with federal and state data protection legislation as well as the General Data Protection Regulation (GDPR).

8 Risk/Benefit Assessment of Study Participation

Based on current evidence, no adverse effects are expected from the administration of questionnaire surveys, qualitative interviews, or focus groups. No risks are known. The diagnostic assessments (e.g. physical examination, blood sampling) are not expected to carry risks beyond those associated with routine clinical care.

The conduct of the new treatment modality (intervention group) as well as the single baseline counselling session (control group) may, due to engagement with sensitive topics (e.g. nutrition, body weight), give rise to transient negative emotional states. These are not specific

to any particular treatment modality and may similarly occur in everyday contexts when confronted with these topics.

No differences in the risk/benefit ratio are expected between the new treatment modality and the single baseline counselling session; the new treatment modality is associated with a greater time commitment compared to the single counselling session, but also with a correspondingly higher expected benefit and, due to greater frequency of contact, a marginally higher associated risk.

The potential risk of excessive media use by participating children and adolescents in the online resources is addressed in the study by: (a) integrating the topic of media literacy—with particular reference to the potential adverse effects of media use in overweight and obese children and adolescents—as an integral content component of the training intervention in the new treatment modality; and (b) restricting online resource use to children aged 12 years and above, with limited use for those aged 13–17 years (in accordance with the recommendations of the Federal Centre for Health Education [BZgA]).

Long-term benefits of this study include the transferability of the concept to regions of Germany beyond Baden-Württemberg, reduced mortality and morbidity in the affected population, reduced morbidity-related costs to healthcare and social systems, and the development of intervention materials that remain usable and transferable in the long term.

9 Outcome Criteria

<u>Primary Outcome Parameters</u>

BMI-SDS_{LMS}

Health-related quality of life (KINDL-R questionnaire)

<u>Secondary Outcome Parameters and Determinants of Treatment Course and Response</u>

Somatic Outcome Parameters
The following parameters are used to identify comorbidities of overweight and obesity in childhood and adolescence and to analyse their course in the intervention group compared to the control group.

Specialist and Physical Examination
Cardiovascular disorders, pubertal development, dermatological conditions, musculoskeletal problems, respiratory tract disorders, fatigue and sleep disturbances (e.g. sleep apnoea)

Sonography
Hepatic disorders

Laboratory Parameters
Total cholesterol, HDL cholesterol, LDL cholesterol, triglycerides, ALT (GPT), fasting glucose, TSH, fT4, HbA1c

<u>Statistical Analyses</u>

The primary analysis is conducted using a frequency comparison with the chi-squared test, with adjustment for the cluster effect (Rao–Scott method). Secondary analyses of the primary endpoint employ logistic regression (with adjustment for relevant baseline covariates and generalised estimating equations [GEE] to account for the cluster effect). Analogous analyses are conducted in the total sample to identify the prognostic value of these covariates. All intervention patients are included in these analyses. Interaction analyses are performed to examine whether subgroups with particularly pronounced treatment effects relative to controls can be identified.

For secondary outcomes, appropriate statistical tests are applied according to scale level and distribution, including the chi-squared test and t-test with adjustment for the cluster structure, as well as more complex regression models (linear, logistic, proportional odds) using the GEE method. All analyses are conducted as intention-to-treat analyses. Efforts will be made to obtain at least the primary endpoint data from participants who drop out of their respective intervention.

In the health economic analyses, the primary endpoints consist of cost-effectiveness calculations for the study treatments and control modalities, and quantification of the financial implications of implementing the study interventions into routine care.

To calculate cost-effectiveness and cost–utility values, standard health economic techniques are applied, including calculation of the incremental cost-effectiveness ratio (ICER), bootstrapping for statistical control of ICER variability, willingness-to-pay estimation, and various sensitivity analyses. The results enable an intervention-, patient-, and setting-independent comparison of cost–benefit ratios against other healthcare interventions across any sector for which comparable parameters (costs per QALY) are available. A subsequent SROI (Social Return on Investment) analysis will additionally model a cross-sectoral, medium- to long-term estimate of the overall societal value generated by the study interventions (e.g. reduction of risk for sequelae such as type 2 diabetes, savings in healthcare costs, reduction in secondary productivity losses among parents, etc.).

10 Data Protection

Standard data protection protocols are applied throughout the study. A comprehensive data protection and IT security concept has been developed and reviewed by the Data Protection Officer of the University Hospital and by the IT Security team. The data protection concept, the affirmative statement of the Data Protection Officer, and the confirmatory statement from IT Security are provided in Appendices 2, 3, and 4. All identified risks have been mitigated through appropriate measures.

11 Appendices

1. Detailed Intervention Description STARKIDS
2. Data Protection and IT Concept (as of 06 April 2020)
3. Statement of the Data Protection Officer
4. Statement of IT Security
5. Participant Information Sheet for Families
6. Informed Consent Form for Families
7. Participant Information for Pediatric Practices
8. Informed Consent Form for Pediatric Practices
9. Current Status of Qualitative Interview Guide Development
10. Participant Information Sheets for Qualitative Assessments (adult and child versions)
11. Informed Consent Forms for Qualitative Assessments (adult and child versions)

12 References

Angermeyer, M. C., Kilian, R., & Matschinger, H. (2000). *WHOQOL-100 und WHOQOL-BREF. Handbuch für die deutschsprachige Version der WHO Instrumente zur Erfassung von Lebensqualitaet.[WHOQOL-100 and WHOQOL-BREF. Handbook of the German Version of the WHO Instrument to Assess Quality of life]*. Göttingen: Hogrefe.

Arbeitsgemeinschaft Adipositas im Kindes- und Jugendalter (AGA), & Deutsche Adipositas-Gesellschaft (DAG), D. G. f. K.-u. J. D. (2019). S3-Leitlinie Therapie und Prävention der Adipositas im Kindes- und Jugendalter Retrieved from https://register.awmf.org/de/leitlinien/detail/050-002

Bammann, K., Reisch, L. A., Pohlabeln, H., Williams, G., & Wolters, M. (2019). Core Questionnaires. In K. Bammann, L. Lissner, I. Pigeot, & W. Ahrens (Eds.), *Instruments for Health Surveys in Children and Adolescents* (pp. 189–207): Springer.

Berger, U., Hentrich, I., Wick, K., Bormann, B., Brix, C., Sowa, M., . . . Strauß, B. (2012). Psychometric quality of the" Eating Attitudes Test"(German version EAT-26D) for measuring disordered eating in pre-adolescents and proposal for a 13-item short version. *Psychotherapie, Psychosomatik, medizinische Psychologie, 62*(6), 223–226. doi: 10.1055/s-0032-1308994

Chen, Y., Porter, K. J., Estabrooks, P. A., & Zoellner, J. (2017). Development and Evaluation of the Sugar-Sweetened Beverages Media Literacy (SSB-ML) Scale and Its Relationship With SSB Consumption. *Health Commun, 32*(10), 1310–1317. doi:10.1080/10410236.2016.1220041

Deci, E. L., & Ryan, R. M. (2000). The" what" and" why" of goal pursuits: Human needs and the self-determination of behavior. *Psychological inquiry, 11*(4), 227–268. doi:10.1207/S15327965PLI1104_01

Diehl, J. M. (1999). Einstellungen zu Essen und Gewicht bei 11-bis 16jõhrigen Adoleszenten. *Schweiz Med Wochenschr, 129*(5), 162–175.

Ebbeling, C. B., Pawlak, D. B., & Ludwig, D. S. (2002). Childhood obesity: Public-health crisis, common sense cure. *The lancet, 360*(9331), 473–482. doi:10.1016/S0140-6736(02)09678-2

Effertz, T., Engel, S., Verheyen, F., & Linder, R. (2016). The costs and consequences of obesity in Germany: A new approach from a prevalence and life-cycle perspective. *The European Journal of Health Economics, 17*, 1141–1158. doi:10.1007/s10198-015-0751-4

Elsayed, H. A. G., Lissner, L., Mehlig, K., Thumann, B. F., Hebestreit, A., Pala, V., . . . Molnár, D. (2020). Relationship between perception of emotional home atmosphere and fruit and vegetable consumption in European adolescents: Results from the I. Family survey. *Public health nutrition, 23*(1), 53–62. doi:10.1017/S1368980019002234

Fichter, M., & Quadflieg, N. (1999). *Strukturiertes Inventar für anorektische und bulimische Eßstörungen:(SIAB); Fragebogen (SIAB-S) und Interview (SIAB-EX) nach DSM-IV und ICD-10; Handanweisung*. Göttingen: Hogrefe, Verlag für Psychologie.

Fliege, H., Rose, M., Arck, P., Levenstein, S., & Klapp, B. F. (2001). Validierung des "Perceived Stress Questionnaire"(PSQ) an einer deutschen Stichprobe. *Diagnostica, 47*(3), 142–152. doi:10.1026//0012-1924.47.3.142

Franzen, S., & Florin, I. (1997). Der Dutch Eating Behavior Questionnaire für Kinder (DEBQ-K)- Ein Fragebogen zur Erfassung gezügelten Essverhaltens *Kindheit und Entwicklung 6*, 116–122.

Fuchs, R. (1997). Soziale Unterstützung und Sportaktivität. In *Psychologie und körperliche Bewegung* (pp. 248–263). Göttingen: Hogrefe.

Fuchs, R., Klaperski, S., Gerber, M., & Seelig, H. (2015). Messung der bewegungs-und sportaktivität mit dem BSA-fragebogen: Eine methodische Zwischenbilanz. *Zeitschrift für Gesundheitspsychologie, 23*(2), 60–76. doi:10.1026/0943-8149/a000137

Giel, K. E., Zipfel, S., Schweizer, R., Braun, R., Ranke, M. B., Binder, G., . . . DISKUS Study Group. (2013). Eating disorder pathology in adolescents participating in a lifestyle intervention for obesity: associations with weight change, general psychopathology and health-related quality of life. *Obesity facts, 6*(4), 307–316. doi:10.1159/000354534

Gut, V., Schmid, J., Schmid, J., & Conzelmann, A. (2019). The Bernese Motive and Goal Inventory for Adolescence and Young Adulthood. *Frontiers in psychology, 9*, 2785. doi:10.3389/fpsyg.2018.02785

Hölling, H., Schlack, R., Kamtsiuris, P., Butschalowsky, H., Schlaud, M., & Kurth, B. (2012). The KiGGS study. Nationwide representative longitudinal and cross-sectional study on the health of children and adolescents within the framework of health monitoring at the Robert Koch Institute. *Bundesgesundheitsblatt, Gesundheitsforschung, Gesundheitsschutz, 55*(6-7), 836. doi:10.1007/s00103-012-1486-3

Jekauc, D., Voelkle, M., Wagner, M. O., Mewes, N., & Woll, A. (2013). Reliability, validity, and measurement invariance of the German version of the physical activity enjoyment scale. *Journal of pediatric psychology, 38*(1), 104–115. doi:10.1093/jpepsy/jss088

Junne, F., Ehehalt, S., Ziser, K., Reinehr, T., Wiegand, S., Mander, J., . . . Wabitsch, M. (2019). Targeting parental motivation for change in childhood obesity: development and validation of the PURICA-S scale. *International Journal of Obesity, 43*(11), 2291–2301. doi:10.1038/s41366-019-0415-z

Kliem, S., Mößle, T., Zenger, M., Strauß, B., Brähler, E., & Hilbert, A. (2016). The eating disorder examination-questionnaire 8: A brief measure of eating disorder psychopathology (EDE-Q8). *International Journal of Eating Disorders, 49*(6), 613–616. doi:10.1002/eat.22487

Kliem, S., Schmidt, R., Vogel, M., Hiemisch, A., Kiess, W., & Hilbert, A. (2017). An 8-item short form of the Eating Disorder Examination-Questionnaire adapted for children (ChEDE-Q8). *International Journal of Eating Disorders, 50*(6), 679–686. doi:10.1002/eat.22658

Kromeyer-Hauschild, K., Moss, A., & Wabitsch, M. (2015). Referenzwerte für den Body-Mass-Index für Kinder, Jugendliche und Erwachsene in Deutschland. Anpassung der AGA-BMI-Referenz im Altersbereich von 15 bis 18 Jahren. [Body mass index reference values for German children, adolescents and adults. Modification of the AGA BMI reference in the age range between 15 and 18 years]. *Adipositas - Ursachen, Folgeerkrankungen, Therapie, 09*(03), 123–127. Retrieved from http://www.thieme-connect.de/products/ejournals/abstract/10.1055/s-0037-1618928. doi:10.1055/s-0037-1618928

Kromeyer-Hauschild, K., Wabitsch, M., Kunze, D., Geller, F., Geiß, H. C., Hesse, V., . . . Korte, W. (2001). Perzentile für den Body-mass-Index für das Kindes-und Jugendalter unter Heranziehung verschiedener deutscher Stichproben. *J Monatsschrift Kinderheilkunde, 149*(8), 807–818.

Kurth, B.-M., & Rosario, A. S. (2007). Die Verbreitung von Übergewicht und Adipositas bei Kindern und Jugendlichen in Deutschland. *Bundesgesundheitsblatt-Gesundheitsforschung-Gesundheitsschutz, 50*(5-6), 736–743. doi:10.1007/s00103-007-0235-5

Latendresse, S. J., Rose, R. J., Viken, R. J., Pulkkinen, L., Kaprio, J., & Dick, D. M. (2009). Parental socialization and adolescents' alcohol use behaviors: Predictive disparities in parents' versus adolescents' perceptions of the parenting environment. *Journal of Clinical Child & Adolescent Psychology, 38*(2), 232–244. doi:10.1080/15374410802698404

Levin-Zamir, D., Lemish, D., & Gofin, R. (2011). Media Health Literacy (MHL): development and measurement of the concept among adolescents. *Health education Research, 26*(2), 323–335. doi:10.1093/her/cyr007

Linder, K., Springer, F., Machann, J., Schick, F., Fritsche, A., Häring, H. U., . . . Binder, G. (2014). Relationships of body composition and liver fat content with insulin resistance in obesity-matched adolescents and adults. *Obesity, 22*(5), 1325–1331. doi:10.1002/oby.20685

Lohbeck, A., Schultheiß, J., Petermann, F., & Petermann, U. (2015). Die deutsche Selbstbeurteilungsversion des Strengths and Difficulties Questionnaire (SDQ-Deu-S). Psychometrische Eigenschaften, Faktorenstruktur und Grenzwerte. *Diagnostica, 61*, 222–235. doi:10.1026/0012-1924/a000153

Mander, J., Wittorf, A., Teufel, M., Schlarb, A., Hautzinger, M., Zipfel, S., & Sammet, I. (2012). Patients with depression, somatoform disorders, and eating disorders on the stages of change: Validation of a short version of the URICA. *Psychotherapy, 49*(4), 519. doi:10.1037/a0029563

Manz, K., Schlack, R., Poethko-Müller, C., Mensink, G. B., Finger, J., Lampert, T., & Group, K. S. (2014). Körperlich-sportliche Aktivität und Nutzung elektronischer Medien im Kindes-und Jugendalter. *Bundesgesundheitsblatt-Gesundheitsforschung-Gesundheitsschutz, 57*(7), 840–848. doi:10.1007/s00103-014-1986-4

Mayring, P. (2010). Qualitative inhaltsanalyse. In M. K. Mey G. (Ed.), *Handbuch qualitative Forschung in der Psychologie* (pp. 601–613): VS Verlag für Sozialwissenschaften

Mensink, G. B., & Burger, M. (2004). What do you eat? Food frequency questionnaire for children and adolescents. *Bundesgesundheitsblatt, Gesundheitsforschung, Gesundheitsschutz, 47*(3), 219–226. doi:10.1007/s00103-003-0794-z

Mensink, G. B., Haftenberger, M., & Thamm, M. (2001). Validity of DISHES 98, a computerised dietary history interview: energy and macronutrient intake. *European Journal of Clinical Nutrition, 55*(6), 409–417. doi:10.1038/sj.ejcn.1601174

Mensink, G. B., Schienkiewitz, A., Haftenberger, M., Lampert, T., Ziese, T., & Scheidt-Nave, C. (2013). Overweight and obesity in Germany. *Bundesgesundheitsblatt-Gesundheitsforschung-Gesundheitsschutz, 56*, 786–794. doi:10.1007/s00103-012-1656-3

Mühlig, Y., Scherag, A., Bickenbach, A., Giesen, U., Holl, R., Holle, R., . . . Moss, A. (2017). A structured, manual-based low-level intervention vs. treatment as usual evaluated in a randomized controlled trial for adolescents with extreme obesity-the STEREO trial. *Obesity facts, 10*(4), 341–352. doi:10.1159/000475717

Mühlig, Y., Wabitsch, M., Moss, A., & Hebebrand, J. (2014). Weight loss in children and adolescents— A systematic review and evaluation of conservative, non-pharmacological obesity treatment programs. *Deutsches Ärzteblatt Int, 111*(48), 818–824. doi:10.3238/arztebl.2014.0818

Nolting, H. D., Krupka, S., Sydow, H., & Tisch, T. (2016). Versorgungsreport Adipositas. Chancen für mehr Gesundheit. In H. Rebscher (Ed.), *Beiträge zur Gesundheitsökonomie und Versorgungsforschung (Band 15)*. Heidelberg: medhochzwei Verlag GmbH.

Paul, T., & Thiel, A. (2005). *Eating Disorder Inventory-2 (EDI-2): deutsche Version*: Hogrefe.

Ravens-Sieberer, U., Ellert, U., & Erhart, M. (2007). Gesundheitsbezogene Lebensqualität von Kindern und Jugendlichen in Deutschland. *Bundesgesundheitsblatt-Gesundheitsforschung-Gesundheitsschutz, 50*(5), 810–818. doi:10.1007/s00103-007-0244-4

Salbach-Andrae, H., Schneider, N., Bürger, A., Pfeiffer, E., Lehmkuhl, U., & Holzhausen, M. (2010). Psychometrische Gütekriterien des Eating Disorder Inventory (EDI-2) bei Jugendlichen. *Zeitschrift für Kinder-und Jugendpsychiatrie und Psychotherapie*. doi:10.1024/1422-4917/a000035

Salize, H. J., & Kilian, R. (2010). *Gesundheitsökonomie in der Psychiatrie: Konzepte, Methoden, Analysen*. Stuttgart: Kohlhammer Verlag.

Schienkiewitz, A., Brettschneider, A.-K., Damerow, S., & Rosario, A. S. (2018). Übergewicht und Adipositas im Kindes-und Jugendalter in Deutschland–Querschnittergebnisse aus KiGGS Welle 2 und Trends.

Schlarb, A., Gulewitsch, M. D., Weltzer, V., Ellert, U., & Enck, P. (2015). Sleep duration and sleep problems in a representative sample of German children and adolescents. *Health, 7*(11). doi:10.4236/health.2015.711154

Seelig, H., & Fuchs, R. (2006). Messung der sport-und bewegungsbezogenen Selbstkonkordanz. *Zeitschrift für Sportpsychologie, 13*(4), 121–139. doi:10.1026/1612-5010.13.4.121

Stratton, P., Lask, J., Bland, J., Nowotny, E., Evans, C., Singh, R., . . . Peppiatt, A. (2014). Detecting therapeutic improvement early in therapy: Validation of the SCORE-15 index of family functioning and change. *Journal of Family Therapy, 36*(1), 3–19. doi:10.1111/1467-6427.12022

Truthmann, J., Mensink, G. B., & Richter, A. (2011). Relative validation of the KiGGS Food Frequency Questionnaire among adolescents in Germany. *Nutrition Journal, 10*(1), 133. doi:10.1186/1475-2891-10-133

World Health Organization. (2021). Obesity and overweight. Retrieved from https://www.who.int/news-room/fact-sheets/detail/obesity-and-overweight
