# Supplementary material for: Effects of an e-health enhanced multicomponent outpatient intervention for children and adolescents with overweight or obesity in routine primary care (STARKIDS): a cluster-randomized controlled trial
Source: Lancet Reg Health Eur. 2026 Oct 14;71:101897. doi: 10.1016/j.lanepe.2026.101897 (PMC13694850; doi:10.1016/j.lanepe.2026.101897)
Supplement: Supplementary file 3 [file mmc2.docx]

This translation in German was submitted by the authors and we reproduce it as supplied. It has not been peer reviewed. Our editorial processes have only been applied to the original abstract in English, which should serve as reference for this manuscript.

**Zusammenfassung**

**Hintergrund:** Adipositas im Kindes- und Jugendalter stellt weltweit eine bedeutende Herausforderung für die öffentliche Gesundheit dar und ist mit einem erhöhten Risiko für zahlreiche Folgeerkrankungen, dem Fortbestehen bis ins Erwachsenenalter sowie einer wachsenden wirtschaftlichen Belastung verbunden. Das STARKIDS-Programm ist eine stufenweise, individuell angepasste, e-health-gestützte, familienorientierte Intervention, die in der Primärversorgung durchgeführt wird. Sie integriert Ernährungsstrategien mit dem Ziel der Reduktion der Energiedichte der Nahrung, die Förderung körperlicher Aktivität, verhaltensbezogene Strategien sowie psychosoziale Unterstützung.

**Methoden:** Ziel war es zu prüfen, ob STARKIDS im Vergleich zur üblichen Versorgung die Gewichtsentwicklung und die Lebensqualität verbessert. Es wurde eine cluster-randomisierte kontrollierte Studie in pädiatrischen bzw. hausärztlichen Familienpraxen durchgeführt, die Kinder/Jugendliche mit Übergewicht/Adipositas einschlossen. Die Praxen wurden stratifiziert und blockrandomisiert entweder STARKIDS oder einer einmaligen strukturierten Beratungssitzung (optimierte Standardversorgung, TAU-O) zugeteilt. Endpunkte wurden zu Studienbeginn sowie nach 12 und 18 Monaten erhoben. Primärer Endpunkt war die Veränderung des BMI-z-Scores der teilnehmenden Kinder und Jugendlichen. Studienregistrierung: Deutsches Register Klinischer Studien, DRKS00022813.

**Ergebnisse:** 89 Praxen wurden cluster-randomisiert (44 Interventions-, 45 TAU-O-Praxen). Zwischen April 2022 und Juli 2023 wurden 379 Familien STARKIDS und 232 Familien TAU-O zugeteilt. 343 bzw. 216 Familien wurden in die Intention-to-treat-Analyse eingeschlossen. Nach 12 Monaten erreichten 110 (32%) Teilnehmende der STARKIDS-Gruppe und 45 (21%) der TAU-O-Gruppe die vordefinierte BMI-z-Score-Reduktion ≥ 0.2 (Risikodifferenz (RD) = 0.11, 95%-KI 0.03–0.20, p=0.0256). Nach 18 Monaten zeigten sich keine Gruppenunterschiede in der BMI-z-Score-Reduktion mehr (p=0.6940). Bei der selbstberichteten gesundheitsbezogenen Lebensqualität der Kinder und Jugendlichen zeigten sich zu keinem der beiden Endpunkte signifikante Unterschiede zwischen den Studienarmen. Bei Eltern und Erziehungsberechtigten zeigte sich nach 18 Monaten ein statistisch signifikanter Gruppenunterschied (−2.10, 95%-KI −4.13 bis −0.06; p=0.044), der jedoch nicht als klinisch bedeutsam eingestuft wurde. Sekundäre Endpunkte zeigten hohe Teilnahmequoten, eine breite Nutzung der digitalen Programmkomponenten sowie insgesamt eine hohe Zufriedenheit mit dem STARKIDS-Programm bei Teilnehmenden und Praxisteams, begleitet von moderaten bis hohen Machbarkeits-Bewertungen durch die Praxen. Es wurden keine behandlungsbezogenen schwerwiegenden unerwünschten Ereignisse berichtet.

**Interpretation:** STARKIDS verbesserte den BMI-z-Score über 12 Monate mit Effekten, die mit intensiveren Programmen vergleichbar sind. Der Gemeinsame Bundesausschuss (G-BA) hat die Überführung von Stufe 1 des Programms in die Regelversorgung für Kinder und Jugendliche mit Adipositas empfohlen. Die Effekte blieben nach 18 Monaten nicht bestehen, was auf die Notwendigkeit von Anpassungen hinweist, z.B. zusätzliche Kontaktstunden zur Festigung der erzielten Ergebnisse, gefolgt von einer schrittweisen Reduktion der Kontaktstunden.

**Förderung:** Innovationsausschuss des Gemeinsamen Bundesausschusses (G-BA)
